# Identifying barriers to the educational role of midwives in Cyprus and defining determinants in behaviour terms using the Behaviour Change Wheel: a mixed-method formative study

**DOI:** 10.1186/s12913-022-08599-7

**Published:** 2022-10-05

**Authors:** Nicos Middleton, Eleni Hadjigeorgiou, Ourania Kolokotroni, Veronika Christodoulides, Ioanna Koliandri, Christiana Nicolaou, Maria Papadopoulou, Christiana Kouta, Maria Karanikola, Alison Baum, the Baby Buddy Forward team, Julia Leinweber, Britta Bachetta, Kleanthi Gourounti, Antigoni Sarantaki, Katerina Lykeridou, Olga Gouni, Shabira Papain, Stephanie Kronson

**Affiliations:** 1 Department of Nursing, School of Health Sciences, Cyprus University of Technology, Limassol, Cyprus; 2 Birth Forward, Non-Governmental Organization, Nicosia, Cyprus; 3 Department of Primary Care and Population Health, University of Nicosia Medical School, Nicosia, Cyprus; 4 Best Beginnings, Registered Charity Organization, London, UK

**Keywords:** Midwives, Antenatal education, Behaviour change, COM-B, Theoretical domains framework, Formative research, Intervention design

## Abstract

**Background:**

Τhe Baby Buddy Cyprus webapp was co-created with parents and health professionals within a Participatory Action Research framework. While using Baby Buddy in routine consultations can support the educational role of mother–child healthcare providers (HP), antenatal education (AE) may be currently perceived as a formal activity within the physical space of the antenatal class. We aimed to gain an understanding of influences on midwives engaging in an educational role during routine appointments and identify potential interventions using the Behaviour Change Wheel (BCW) framework.

**Methods:**

This is a formative mixed-methods research study, with a convergent parallel design, guided by the COM-B model and related Theoretical Domains Framework (TDF). Complimentary methods were used to collect information from in-training and registered midwives: focus group (*N* = 11), questionnaire survey (*N* = 24) and Nominal Group Technique during workshops (*N* = 40). Deductive content analysis of qualitative data and quantitative survey analysis shaped the behaviour diagnosis along the 6 COM-B and 14 TDF domains, and informed the selection of relevant intervention functions and related Behaviour Change Techniques from the BCW taxonomy.

**Results:**

AE is viewed as a core function of the professional role, yet neither supported nor prioritized by current practices. Problematic areas relate to organizational context, such as weak interprofessional collaboration and lack of policy, protocols and resources. In addition, medicalization of birth and related socio-cultural norms, pertaining to users and providers, are sustaining alienation of the midwife and conditions of power dynamics. AE was perceived as a means to enhance the autonomy of the profession but there might be issues with procedural knowledge and the need for skill development was identified. Several intervention functions were identified as promising, however cognitive re-framing through strategic communication and modelling may also be needed both in terms of providing “credible models” for the role itself as well as re-framing AE through the concept of “making every contact count”.

**Conclusions:**

AE is currently perceived to be a ‘bad fit’ with routine practice. The study identified several barriers to the educational role of midwives, influencing Capacity, Opportunity and Motivation. While digital tools, such as Baby Buddy, can facilitate aspects of the process, a much wider behaviour and system change intervention is needed to enhance midwives’ educational role and professional identity. In addition to proposing a theory-driven research-informed intervention, the process functioned as a participatory learning experience through collective reflection.

**Supplementary Information:**

The online version contains supplementary material available at 10.1186/s12913-022-08599-7.

## Contribution to the literature

- Digital tools and resources have the potential to transform antenatal education but only if adopted by healthcare providers as part of everyday clinical practice.
- Several barriers to being an effective educator have been described; yet, previous studies do not always incorporate an explicit process of translating the findings into designing an intervention to affect change.
- This study describes all the stages of the Behaviour Change Wheel process from using mixed-methods to diagnose the issue among midwives in Cyprus to intervention mapping.
- Guided by Participatory Action Research, the process was also instrumental in cultivating a new shared awareness of the issue.

## Background

The transition to parenthood is a “*window of opportunity*” for the establishment of health-promoting behaviours. The goal of antenatal education (AE) is to support expectant parents through this life-changing event [1, 2]. “Traditional” approaches to AE include structured programmes, of various duration and content, often in group sessions, mainly focusing on preparation for childbirth. Attendance is not always high; in Cyprus, only one in three women attend classes [3, 4]. Furthermore, their effectiveness has been questioned in terms of whether they address the real needs of expectant parents [5–8] and their inclusiveness, with evidence of inequalities in access and outcomes [9–11].

While every contact with a healthcare provider can be a “*teachable moment*” [12], these encounters may not be as rewarding and the experience can be dependent on the quality of the user-provider communication [13, 14]. The WHO recommendations (2016) for a positive pregnancy experience and optimal maternal and newborn health refer to “*Respectful Maternity Care*” [15]. The framework recognizes informational and emotional support as a prerequisite, provided “*by knowledgeable, supportive and respectful health-care practitioners with good clinical and interpersonal skills within a well-functioning health system*”. A range of reasons have been previously described as to why healthcare providers may not be actively engaging with expectant parents in an educational capacity during appointments, such as time and resource constraints but also lack of confidence in skills and competences [16–18].

New technologies are playing an increasingly important role and digital innovation can assist in the education role [19]. Baby Buddy Forward is a Participatory Action Research programme which assessed the cross-cultural transferability of the Baby Buddy app from the UK to Cyprus. The Baby Buddy Cyprus webapp, with rich material from early pregnancy to the first six months of the baby’s life and a library of over 200 short videos, was co-created through an iterative approach with the participation of over 800 parents-to-be and health professionals. Other than widening opportunities to evidence-based information, Baby Buddy offers a complimentary tool to assist healthcare providers in their educational role. However, the impact of digital technologies can only be maximized when healthcare providers engage with service-users in an educational capacity. While actively embedding Baby Buddy in clinical practice was beyond the scope and timeline of the project, it was important to identify in a systematic and comprehensive manner the various components necessary to affect this change. While the issue concerns all maternal-child healthcare providers, AE is a core part of midwives’ professional role.

## Aim

This formative study aims to (a) gain an understanding as to why midwives in Cyprus may not be actively engaging in an educational capacity during routine appointments, defining them in behaviour terms using the COM-B and related Theoretical Domain Framework (phase 1) and (b) map identified determinants to a potential intervention using the Behaviour Change Wheel process and related taxonomy of intervention functions and Behaviour Change Techniques (phase 2). Prior to the primary research, a rapid review was performed to identify studies in the international literature that explored barriers to midwives’ educational role using the COM-B and/or TDF to collect and/or analyze data (preparatory phase).

This study is positioned within a pragmatic research paradigm both in terms of the formative nature of the research question (meaningful identification of intervention components within a specific organizational context) as well as the mixing of different research traditions [20]. According to Kelly and Cordeiro (2020), pragmatism is a good fit for research in organizational contexts since, as an epistemology, to quote: “*is premised on the idea that research can steer clear of metaphysical debates about the nature of truth and reality and focus instead on ‘practical understanding’ of concrete, real-world issues*” [21]. Three central tenets in the pragmatic inquiry [21], are: (1) emphasis on actionable knowledge (in this case, identification of influences, among a pre-determined set of determinants, upon midwives engaging with a particular behaviour in their professional capacity), (2) interconnectedness of experience, knowing and acting (i.e. translating the experience to a proposed intervention) and (3) inquiry as an experiential process (i.e. incorporating elements of participatory learning in the research process to promote collective reflection among those impacted by the organizational processes).

## Methods

### Study design

The implementation of the Behaviour Change Wheel approach is flexible. A range of primary and secondary research methods can be utilized in the process of behaviour diagnosis, whereas the intervention design stage may take the form of an internal process, or, more powerfully, incorporate elements of co-production, directly involving the target group [22]. A mixed-method approach with a convergent parallel design was adopted as more appropriate for the purpose of addressing the first study aim (i.e. define barriers to the educational role of midwives in Cyprus in behaviour terms). Thus, for phase 1, the study reports on three complementary sources of information and informants: focus group with in-training midwives (*study A*), questionnaire survey with Midwifery leadership (*study B*) and participatory learning workshop with practicing Registered Midwives (*study C*) – described in more detail below.

In all three, the COM-B model and/or Theoretical Domains Framework (TDF) were adopted for informing both data collection and analysis. All three studies were deemed necessary in providing an understanding of the target behaviour, with no set priority or sequence, while neither methodological approach limits or confounds the implementation or interpretation of the other. Furthermore, it was necessary to target different groups, thus, matching the method to the participants and setting accordingly. Each study was analysed separately and, as appropriate for convergent designs, mixing occurred at the interpretation stage to provide a unified narrative of the findings.

### COM-B, TDF and Behaviour Change Wheel

The Behaviour Change Wheel (BCW) [22] offers a structured process of designing an intervention (*second phase*) based on what is understood about the target behaviour (*first phase*). At the core is the COM-B model, recognizing Capability (C), Opportunity (O) and Motivation (M) as an interacting system of determinants of Behaviour (B). Capability is further defined as physical and psychological, Opportunity, as physical and social, and Motivation, as automatic and reflective. The process of defining influences on the behaviour of interest (*“not engaging in an education capacity during routine appointments”*) in behaviour terms can be enhanced by the more detailed Theoretical Domains Framework (TDF) The components of the two models and their relationship are presented in Additional File 1 [23].

The two outer layers of the wheel refer to nine intervention functions that can be used to affect behaviour (namely: Education, Persuasion, Incentivisation, Coercion, Training, Enablement, Modelling, Environmental Restructuring and Restrictions) and seven policy categories to support delivery (namely, Guidelines, Environmental planning, Service provision, Communications and Marketing, Fiscal measures, Regulation and Legislation). Specific Behaviour Chance Techniques (BCTs), considered ‘active ingredients’ of the intervention, can be selected from a taxonomy of 93 BCTs in 16 categories, and an app has been developed to facilitate the process [24].

### Implementation process

Implementation of the BCW process involves completing a series of tasks, namely 1. Specify target behaviour, 2. Conduct behaviour analysis, 3. Identify potential intervention functions, 4. Identify policy categories to support the intervention functions, 5. Select BCTs to deliver intervention functions, and 6. Use APEASE criteria to assess Appropriateness, Practicability, Effectiveness, Acceptability, Side effects and Equity of selected intervention functions. Tasks 1 and 2 form the original research part of this process (‘behaviour diagnosis’, referred here as phase 1) which informed the intervention design. In order to frame the method, findings and proposed intervention in the context of the literature, a rapid review was also performed to identify similar studies among midwives internationally (*preparatory phase*).

**Fig. 1:**
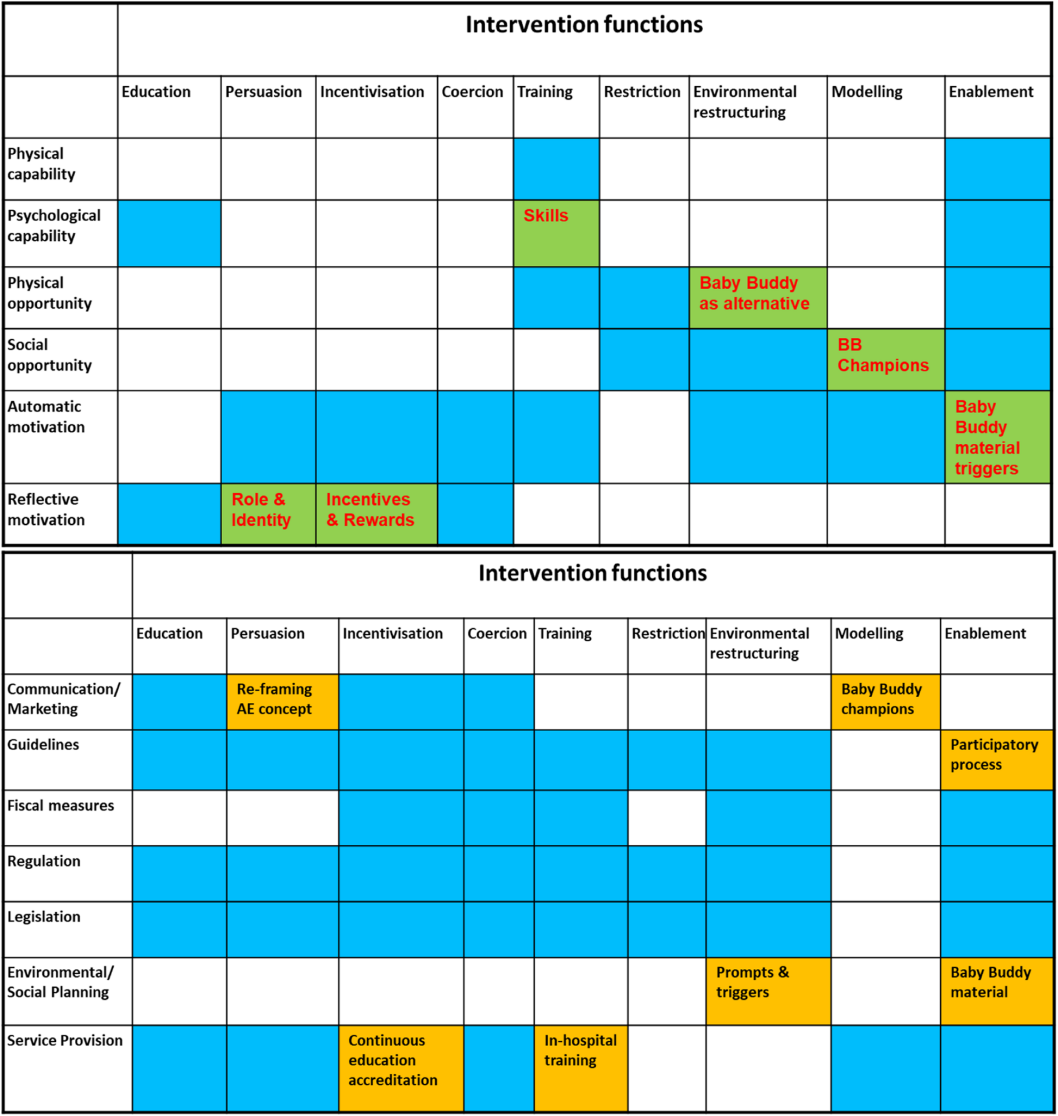
Matrices of selected intervention functions and related policy categories

Tasks 3–6 concern the intervention design (phase 2). This mapping-exercise was performed by the Baby Buddy Forward core team, during a two-day workshop, after formal training in the implementation of the BCW. The BCW guide, developed by the UCL Centre of Behaviour Change, with related tools and worksheets were used in the process. After presenting the ‘behaviour diagnosis’, workshop participants were asked to reflect on the findings and suggest appropriate intervention functions, while considering the issues and/or suggestions of the workshop participants (study C). They were also asked to incorporate the Baby Buddy webapp in their suggestions, if possible. Each participant was provided with a blank sheet of the BCW matrices, linking: (1) COM-B domains to intervention functions and (2) intervention functions to relevant policy categories (see Fig. 1). In a round-robin method, all suggestions (broad or specific) were written on a whiteboard. Their justification was discussed and debated, arriving at recommendations by consensus. Once mapping was completed, relevant BCTs were identified based on the core justification provided by the team with regard to the actual behaviour component the intervention function is purported to address. A proposed list was compiled along each intervention function but, due to the plurality of the suggestions, a detailed assessment of each on the basis of the APEASE criteria was avoided.

Methodology reporting standards were observed, both for reporting qualitative studies (COREQ) and for reporting implementation studies (StaRI checklist)—Additional file 2. A descriptive account of the transparency criteria for replicability, as described by Aguinis and Solarino (2019) is also provided [25]. Empirical replication of the findings in a different population and setting might be less relevant due to the socio-political specificity of the issue; thus, information about the current state of Midwifery in Cyprus and the relationship of researchers with participants and clinics were incorporated in the account.

### Preparatory phase: rapid literature review

The search strategy, inclusion/exclusion criteria as well as characteristics of identified studies are presented in Additional File 5. Due to the context-specific nature of the issue, the focus was less on the actual findings of these studies and more on (a) the study design, range of methods and, more importantly, tools employed by previous studies in the interest of consistency and (b) the process of mapping the ‘behaviour diagnosis’ to a proposed intervention. Studies were considered even if they did not present all the stages of the BCW process. In fact, as shown in the Table (Additional File 5), among 15 identified studies, only three included some process of mapping findings to intervention functions and/or BCTs, whereas the rest only made generic recommendations in the discussion section of the article. Furthermore, only three studies employed mixed-methods with the majority opting for a qualitative or a quantitative approach, adopting either the COM-B or the TDF model. None of the identified studies used both frameworks in parallel. In addition to identifying appropriate tools (topic guide and questionnaire) to use in this study, this observation reinforced the decision to employ a mixed-method approach, since the simplicity of the COM-B is a better fit for a qualitative approach or participatory learning activities, while of a quantitative survey is more apt for the more detailed TDF.

### Study A: focus groups and written response to open-ended question

Postgraduate midwifery students in their final semester prior to registration (*N* = 11) were provided with a statement regarding “teachable moments” and an open question on factors that facilitate or present obstacles for a midwife to actively engage in an educational capacity during appointments. The process was followed by a two-hour focus group session. The online session, started with a brief presentation of the initial findings, allowing researchers to confirm the original classification and giving participants the opportunity to provide feedback, elaborate on their responses or raise additional suggestions. A topic guide, adapted from previous studies [26–28] was used to structure the discussion and discriminate between the more detailed TDF classification (Additional File 3). Transcripts were thematically analysed according to the Framework method [29, 30], which operates from a pragmatic epistemology. Furthermore, the method lends itself easily to policy and practice-oriented questions and facilitates, due to its clear structure and boundaries, triangulation with other methods. Deductive (directed) content analysis was used to code the written responses using the COM-B and TDF as an *a priori* framework of categories, with codes developed iteratively based on the operational definition of each domain and related concepts. Codes were used to index and chart the transcripts accordingly along the COM-B and TDF model. The unit of analysis was short phrases, as a full sentence may contain elements pertaining to different domains. The transcripts were read several times to ensure familiarity and coded independently by two researchers (NM, public health/ health services researcher and EH, academic midwife), who discussed and debated their classification regularly to identify any discrepancies in the coding and ensure process transparency. Meaningful text which could be attributed to more than one domain was coded as such, and a decision was made by consensus in terms of the most appropriate domain. Where necessary, any disagreement in terms of the classification was resolved by involving a third researcher (MK, academic nurse). While inductive processes may have generally allow a fuller exploration of the phenomenon, opting for a deductive manifest, rather than latent, analysis was a better fit with the research paradigm, the data collection approach (written responses and focus group discussions vs richer descriptions from personal interviews) and purpose of the study (formative research for proposing an intervention vs in-depth understanding of the experiences of midwives in their role as antenatal educator) [31].

### Study B: questionnaire survey

A questionnaire survey was distributed online to a purposive sample representing the current leadership of the Midwives Committee of the Cyprus Nurses and Midwives Association (N = 24, 38% with more than 20 years of experience). A generic TDF tool, referring to A = Action, C = context, T = time, and Ta = target was used with permission from the developers [32–34]. The tool was adapted accordingly to refer to “antenatal education and counseling” (A) during routine antenatal appointments (C, T) with pregnant women and partners (Ta). The original versions consist of 79–93 items [26, 27] and has been extensively used in similar studies since it is one of the few tools that have been shown to tap on all TDF factors. Based on a process of assessing the discriminant validity of the items, a shorter 38-item version was proposed [32], which nevertheless does not cover all TDF domains. After comparing the three versions, an 83-item tool was compiled. This included all 38 items with good discriminatory validity as well as additional items sourced, upon assessing their face validity, from the two longer versions in order to cover all 14 TDF domains and 24 sub-domains (see Additional File 4, along with the Greek translation). A forward–backward translation process was used to retain semantic equivalence. Each item is measured on a 6-point Likert response scale (Totally Disagree-Totally Agree, or similar depending on question). Due to the length of the questionnaire, items were grouped based on similar wording and introduced as sub-items around a common stem. Average scores per item as well as scores along the 24 TDF sub-domains were calculated. The small sample size, especially given the length of the tool, did not permit formal validation, beyond face validity. However, in the first instance, the analysis focused on ranking the domains, as a rough indication of areas considered by the leadership of Midwifery as more and less problematic in relation to the antenatal education role.

### Study C: participatory learning workshop

The launch of Baby Buddy provided an opportunity for a participatory learning activity around challenges faced by registered midwives (RM) in performing their educational role. This was an open event, which attracted over 120 delegates (about one in three of the midwifery workforce). Participation in the event was accredited with Continuous Education Credits, a necessary requirement for renewal of registration for Nurses and Midwives in Cyprus every four years. A workshop was organised with a sub-set of delegates (*N* = 40) in six parallel groups due to room size restrictions related to COVID-19. Participants were Registered Midwives (RM) from all five state hospitals and most private clinics across all districts of the island, with working experience spanning from newly registered to over 30 years. After a short presentation, each 6–7-member group was given 30 min to reflect as a team and record their collective experience regarding challenges and potential opportunities on an A3 worksheet, divided in three colour-coded sections along the COM-B dimensions. A modified Nominal Group Technique approach was used to structure the discussion while giving all teams the chance to contribute. In a round-robin session, suggestions were written on a whiteboard and the process continued until there were no more suggestions. If a suggestion was similar to a previous point, the group would decide whether it merited to be mentioned as distinct. The process did not include voting and ranking since the purpose was to collect as many suggestions as possible. Other than the classification along the rough dimensions of Capacity, Opportunity and Motivation, as proposed by the participants during the workshop, the process did not entail further analysis, other than grouping similar/related suggestions, linguistic/syntactical editing and translating in English. Often participants found it difficult to classify their suggestions according to COM. With the assistance of trained facilitators (NM, EH, VC, CN, MP, CK), the group would discuss, debate and decide which domain to best assign it to, with the understanding that some factors represent horizontal themes.

### Ethical considerations

The study was performed in accordance with the Declaration of Helsinki and has been approved by the Cyprus National Bioethics Committee as part of the wider Baby Buddy Forward programme. Before participating in face-to-face sessions or completing the questionnaire, the purpose and process of data collection and analysis were explained to the participants who provided informed consent. Confidentiality of the information provided in face-to-face sessions was maintained by anonymization when presenting quotes. With regard to the survey, participation was anonymous and no personal data was recorded.

## Results

Table 1 presents a count summary of the content analysis of study A, along with example quotes across the COM-B and TDF. The number of participants referring to each of the 14 TDF dimensions as well as the frequency of references serve as indicators of completeness. Adopting, in parallel, a quantitative content analysis approach is a good fit with framework analysis. It is a common approach in nursing and health services research [35], and a frequent feature in similar studies. However, it should be noted that this process was undertaken mainly as a means to (a) visually display the data along the COM-B and TDF framework matrix and (b) facilitate method triangulation with the survey results. For instance, “Environmental Context and Resources” was the domain with the highest frequency of quotes. Consistently, this was the domain with the lowest score in the survey, especially with regard to resources, policy, training opportunities and other support mechanisms. However, irrespective of the frequency, all contributions by participants were considered insightful and of equal value. This is reflected in the narrative account covering all COM-B/TDF and were given due consideration in selecting thecomponents of the proposed intervention.

**Table 1:** Frequency of quotes (intensity) and participants referring to each domain (count) classified according to COM-B and TDF

**Theoretical Domain Framework**	**COM-B**	**Participants**											**Count**	**Intensity**	**Characteristic quote**
		**1**	**2**	**3**	**4**	**5**	**6**	**7**	**8**	**9**	**10**	**11**			
**(1) Skills (physical)**	**Physical Capability**												0	0	
**Skills (cognitive & interpersonal)**	**Psychological Capability**	√		√				√	√	√	√	√	7	9	“The **skills** needed by a midwife who works in the maternity ward, and operates within routine practice, are different from the ones needed by a midwife working with pregnant women [re: antenatal clinic]”
**(2) Knowledge**		√		√				√	√	√	√	√			"I noticed that each midwife offers different ‘knowledge' [*re: information*], resulting in confusion and misinformation. This is done either….or … or due to actual lack of **knowledge**”
**(3) Memory, attention & decision process**		√				√				√			3	3	"…small number of midwives … As a result, a midwife is often **unable to devote** as much time as needed to a pregnant woman”
**(4) Behavioural regulation**				√					√				2	2	"Factors which can facilitate the educational role of the midwife are…., (2) **planning** of midwifery care, (3) cooperation with other health professionals, (4)…."
**(5) Environmental context & Resources**	**Physical Opportunity**	√	√	√	√	√	√	√	√	√	√		10	18	“Personal contact presupposes … a suitable space with a desk and a computer, comfortable chairs, beautiful, friendly **environment**…”, “…available, accessible and good-quality educational material and services… there is insufficient **resources**…"
**(6) Social influences**	**Social Opportunity**		√	√	√	√		√	√				6	9	"…the **medicalization** of the birth but also the **mentality** of many Cypriots who always seek the opinion of the doctor"
**(7) Social or Professional Role & Identity**	**Reflective Motivation**			√					√		√	√	4	8	“A midwife has a **duty** to provide equal care to all without discrimination…” “…midwives work with significant limitations in terms of their **autonomy**”
**(8) Beliefs about Capabilities**				√				√					2	3	“The midwife **has accepted** this way of working, to monitor the pregnant women only at the time of childbirth… as a result she does not train more, does not specialize, does not take initiative and does not assume her educational role beyond the moment of childbirth and for breastfeeding support”
**(9) Beliefs about Consequences**			√			√	√	√	√				5	6	"[A midwife] has an important role to play with regard to counselling and education, not only for the **women but also for the whole family, as well as for the community**. Her work includes prenatal education and preparation for parenting but can be extended to women's health, sexual and reproductive health, and pediatric care”
**(10) Optimism**								√			√	√	3	3	“Today's midwives must have the **courage** [to dare] to showcase their abilities in order to promote their autonomy as midwives.”
**(11) Intentions**								√		√			2	2	“…**indifference** on the part of midwives” “…it depends on the **personal will**, desire and time each midwife has”
**(12) Goals**									√	√	√		3	3	"… the most important [facilitating] factor is to have or be able to build a **relationship based on trust** between the pregnant woman and the midwife"
**(13) Reinforcement**	**Automatic Motivation**			√		√	√	√	√	√			6	7	“…there isn’t any **common code of practice** or guidelines, which if existed the problem would be resolved.”
**(14) Emotion**										√	√	√	3	5	“…increases the **job satisfaction** and enhances professional autonomy and responsibility in the workplace”

Table 2 presents survey scores (study B) along the 24 TDF sub-domains, reversed ranked from more to less problematic. The most problematic areas related to lack of organizational resources and support from management. In contrast, “Social and Professional Role and Identity” along with “Beliefs about Consequences” (positive attitudes and outcome expectancies) received the highest scores. This was largely consistent with qualitative descriptions about the importance and value midwives ascribe to AE as well as how they view it as a core function of their professional role, irrespective of the role not being a “good fit” within the current socio-political context Interestingly, while “Intentions” to perform this role also scored high, achieving “Goals” received one of the lowest scores. This appears to be mainly due to perceived competing priorities, which was also described by participants in the other two studies. However, in terms of procedural knowledge and skills to perform the role, some important discrepancies were identified between the survey and the other two studies. While in the survey (midwives’ leadership), knowledge and skills scored high, focus group (in-training midwives) and workshop participants (RM) identified significant knowledge gaps and clearly identified the need for skills strengthening training. In fact, the workshop was instrumental in delineating particular areas that need attention (e.g. communication, critical appraisal and digital skills) and preferred mode of delivery (e.g. in-hospital experiential training).

**Table 2:** Scores and rank of more to less problematic aspects classified by TDF domains (*N* = 14) and sub-domains (*N* = 24)

**Domains (*N* = 14)**	**Items (*N* = 83)**	**Sub-domains (*N* = 24)**	**6-point Likert disagreement agreement scale**		**Sub-domain score (theor. range 1–6)**		**Rank (reverse)**
			**Mean**	**SD**	**Mean**	**SD**	
Knowledge	Aware	Procedural knowledge & Role clarity (4 items)	5.46	0.78	5.43	0.60	20
	Know		5.54	0.66			
	Familiar		5.25	0.94			
	Expected (role clarity)		5.46	0.78			
Skills	Trained	Skills (4 items)	5.50	0.59	5.34	0.56	19
	Skills		5.38	0.71			
	Practiced		5.25	0.85			
	Proficiency		5.25	0.74			
Social/professional role and identity	Part of work	Professional role (4 items)	5.71	0.55	5.73	0.43	24
	Job as midwife		5.71	0.55			
	Professional Responsibility		5.75	0.44			
	Consistent with profession		5.75	0.53			
Beliefs about capabilities	Confident – even if participants not motivated	Self-efficacy (3 items)	5.04	0.81	5.28	0.63	17
	Confident – when little time		5.25	0.85			
	Self-confident		5.54	0.66			
	Control	Perceived behavioural control (3 items)	4.38	1.41	4.81	0.83	10
	Difficult-Easy		4.92	0.88			
	Impossible-Possible		5.13	0.95			
Optimism	Expect best in uncertain times	Optimism (3 items)	5.13	0.99	5.32	0.66	18
	Optimistic about the future		5.46	0.66			
	Expect more good than bad		5.38	0.71			
Beliefs about consequences	Benefit mother–child health	Outcome expectancies (5 items)	5.79	0.41	5.43	0.48	21
	Benefit public health		5.67	0.56			
	Disadvantages for relationship (*reverse*)		4.54	1.38			
	Satisfaction		5.63	0.58			
	Collaboration with professionals		5.54	0.72			
	Useless – useful	Attitudes (3 items)	5.58	1.10	5.72	0.57	23
	Bad – Good		5.79	0.51			
	Not worthwhile—Worthwhile		5.79	0.51			
Reinforcement	Financial reimbursement	Reinforcement (4 items)	2.38	1.58	4.49	0.65	5
	Recognition from peers		4.71	1.12			
	Make a difference		5.21	0.88			
	Recognition from participants		5.67	0.48			
Intentions	Next 10 appointments	Intention (4 items)	5.42	0.95	5.63	0.52	22
	Determination		5.54	0.72			
	Intention		5.75	0.53			
	Strength of intention		5.79	0.51			
Goals	Clear plan—Process	Priority (4 items)	5.17	0.76	3.98	0.67	4
	Clear plan—Frequency		4.88	0.80			
	Higher priority (*reverse*)		2.79	1.25			
	More urgent (*reverse*)		3.08	1.02			
Memory, attention and decision processes	Easy to remember	Memory and Attention (5 items)	4.92	0.93	4.76	0.70	9
	Forget		4.96	1.23			
	Concentration		5.00	1.14			
	Distracting thoughts (*reverse*)		4.21	1.44			
	Attention focus		4.71	1.04			
Environmental context and resources	Institutional financial support	Characteristic of socio-political context (4 items)	2.79	1.32	3.65	1.14	3
	Good networks between parties		3.79	1.28			
	Fit with routine practice		4.13	1.45			
	Routine in organization		3.88	1.54			
	Enough time	Organizational resources (6 items)	3.50	1.32	3.31	0.99	1
	Professional training		3.96	1.37			
	Necessary resources		3.04	1.37			
	Financial reimbursement		2.33	1.20			
	Sufficient material		3.75	1.39			
	Assistance		3.25	1.39			
	Motivation of participants	Characteristics of participants (2 items)	4.83	1.13	4.90	1.12	12
	Positive response from participants		4.96	1.16			
Social influences	Peer acceptance	Subjective norm (2 items)	5.17	0.87	5.17	0.86	14
	Peer approval		5.17	0.87			
	Rely on team of professionals	Social support (4 items)	4.42	1.28	4.57	0.99	7
	Colleagues are willing to listen		4.63	1.17			
	Helpful team of professionals		4.50	1.18			
	Rely on colleagues		4.75	1.03			
	Team of professionals do it	Descriptive norm (2 items)	4.46	1.22	4.56	1.07	6
	Respected colleagues do it		4.67	1.17			
	Management support	Organizational support (3 items)	3.46	1.41	3.53	1.39	2
	Management willing to listen		3.46	1.44			
	Helpful management		3.67	1.40			
Emotion	Enjoy normal day-to-day activities	Stress (2 items)	4.83	1.05	4.63	0.85	8
	Unhappy and depressed (*reverse*)		4.42	1.21			
	Inspired	Affect: Positive and negative emotions (2 items)	5.42	0.65	5.04	0.79	13
	Nervous (*reverse*)		4.67	1.27			
Behavioural regulation	Automatically	Automaticity (4 items)	5.29	1.04	5.21	0.81	16
	Without thinking		5.13	0.99			
	Without having to consciously remember		5.29	1.00			
	Start before realize doing it		5.13	1.36			
	Track of overall progress	Self-monitoring (3 items)	5.21	0.88	5.18	0.77	15
	Aware of day-to-day behaviour		5.00	0.78			
	Notice successes		5.33	0.87			
	Planning—when participants not motivated	Coping planning (3 items)	4.71	1.27	4.88	0.82	11
	Planning -when little time		4.67	1.09			
	Planning – even if others do not do this		5.25	0.61			

*Legend*: The various TDF sub-domains (*N* = 24) are ranked in reverse order based on mean scores to reflect in ascending order more to less problematic areas related to midwives’ educational role

Finally, Table 3 lists all influences as classified according to the COM-B during the workshop (study C). These assisted in contextualizing and interpreting findings from the other two studies. They provided a nuanced understanding of the issues and offered insights into the nature and sources of the challenges. In fact, they often pertained to ‘areas of action’ with tangible suggestions, which informed the selection of relevant intervention functions. For instance, while medicalization of birth was identified as an important factor limiting the autonomy of midwives by focus group participants, it became clear during the workshop that this is a core horizontal theme, which does not only influence the public acceptance of midwives (hence, limiting their Social Opportunity to engage effectively in AE) but it also directly relates to Capability (e.g. low staffing levels and work overload as a result of midwives being undervalued and underused in the current healthcare system) and Motivation (for instance, through conscious avoidance of the educational role for fear of conflict with doctors in the absence of clear role boundaries, protocols, guidelines or referral systems and, by default, weak interprofessional collaboration).

**Table 3:** Influences on midwives’ educational role classified according to Capability, Opportunity and Motivation during participatory learning workshop (*N* = 40)

**Capability**	**Opportunity**	**Motivation**
**• Continuous education** and professional development: seminars, workshops, conferences, specialised training programmes **• In-hospital training:** absence of formal mechanism for transmission of new knowledge and integration of professional development activities in clinical practice **•** Lack of targeted training in the **educational role** for the development of practical skills **•** Lack of **specialist role** with training in antenatal education and counselling **• Digital skills:** using online and other digital resources or new technologies **•** Strengthening **communication skills** **• Cultural competence** skills needed in changing multicultural environment **• Critical appraisal** skills to keep up-to-date with evidence-based practice **• Evidence-based practice** skills: Ability to refer to and use **evidence** in the context of educational capacity	**•** Inappropriate **infrastructure** / unfriendly spaces, not offering comfort and privacy **•** Lack of **resources:** **•** Lack of educational and informational **material** and other resources **•** Insufficient or non-existent **financial resources** for the creation of material, even if intention exists **•** Lack of **equipment** (e.g. computers) or old-age with no regular maintenance **•** No access to **internet** and online sources of information in clinical settings **• Barriers to access:** **•** Minimal contact with midwives before childbirth (especially in private sector) **• Not institutionalization** of educational role of midwives by organizations / healthcare system **•** Need for establishment of **autonomous** and complementary role **• Direct access** to midwives without referral from an Obstetrician -Gynecologist within new National Healthcre system, even if limited **• Medicalization:** **• Understaffing** and lack of **time** **• Undervalued**: non-recognition of the importance of antenatal education by administration / healthcare system vs midwifery-led care) **•** Professional role **boundaries** and inter-professional **conflict** **•** Lack of common **policy** and **protocols** (roles, actions, referrals, etc.) **• Interdisciplinarty/ interprofessional** activities: **•** Weak **interdisciplinary** collaboration in clinical practice **•** Incompatible **interprofessional** activities, values and skills **•** Need for tighter collaboration and **partnerships** between professional associations and scientific bodies **•** Limited availability of **joint training** programs with other healthcare professionals **• Linguistic barriers:** Inability to communicate with non-Greek healthcare users/ no translators / little to no material in other languages	**•** Competing **priorities** due to work overload / reduced **control** over decision making **•** Inefficient **human resources** utilization **• Professional status and identity:** Unfamiliarity and lack of trust in midwives due to medicalization of birth **• No continuity of care:** Fragmented contact with midwife reduces motivation **• Decentralization**: different roles and responsibilities between state and private maternity hospitals **•** Educational role as a **means** for **•** promoting and showcasing **autonomous role** of midwives **•** improving **job satisfaction** - **•** Strengthening **inter-professional cooperation** in the context of developing guidelines, protocols and material **• Avoidance** of educational role **•** conflicting views with prevailing **medicalised care** **•** i**ncompatible policies** and contradictory messages e.g. maternity leave less than 6 months vs recommendations for exclusive breastfeeding up to 6 months **• Resistance** to change/ **complacency** due to civil servant status in public sector **•** No **continuity** of care –No **community** midwifery **•** Lack of **incentives, recognition or rewards** for professional development **• Unequal opportunities** for professional development **•** No formal **evaluation system** for career progression **•** No wider **culture of continuing education** and professional development **•** Establishment of first midwifery-led natural **birth centre** reason for optimism

An integrated account from all three primary studies follows, structured along the COM-B domains. To support contextualization, the narrative is best illustrated with quotes from study A, while references to the other two studies appear in parentheses as integrated in the narrative to provide a nuanced understanding of the issue and/or further support credibility of interpretation.

### Phase 1: behaviour diagnosis

#### Physical Opportunity

Many of the problematic areas relate to the Context where the activity takes place and lack of Resources (A, B, C). Understaffing, heavy workload, lack of time and competing priorities do not present opportunities for a midwife to engage effectively in an educational role:

- Participant 2: *“…small number of midwives … As a result, a midwife is often unable to devote as much time as needed to a pregnant woman”.*

In addition, unwelcoming spaces and other physical barriers act as additional stressors (A, C). Often, the activity takes place in uncomfortable settings or situations due to lack of dedicated spaces (C). This person-environment interaction stressors raise issues of privacy.

- Participant 9: *“…midwives meet 2 or even 3 women in the same room at the same time without any privacy****…****”.*
- Participant 1*: “Personal contact presupposes the availability of time for discussion and questions, but also a suitable space with a desk and a computer, comfortable chairs, a beautiful, friendly environment…”*.

Participants referred to lack of material (e.g. leaflets), equipment (e.g. computers), facilities (e.g. internet access) and other necessary resources (A, C). Weak institutional financial support prevents from developing material, even if the initiative exists (B, C). There are also linguistic barriers with service-users who are non-Greek speakers (A, C).

- Participant 1: *“…available, accessible and good quality educational material and services …there isn’t sufficient material and resources, …"*.
- Participant 6: *“…the issue of language and communication is an obstacle … a large proportion of women are from abroad … material should be translated and antenatal classes should be provided in other languages or there should be a translator available”*

More importantly, the fact that the educational role of the midwife is not institutionalized by the system is presenting barriers to non-fragmented access to the women (C). In fact, the opportunity that the system affords the midwife is significantly diminished in the private sector, where the midwife might not have any meaningful contact with the woman before labour (A, C).

- Participant 7: *“…all pregnant women are monitored by the doctor, midwives in hospitals see them for a minute or two for vital signs and in private clinics women do not even come in contact with the midwife, only the doctor”.*

The current model of healthcare delivery directly reduces the opportunity for meaningful contact, but also acts as a deterrent by impacting motivation (A, C).

- *Participant 9: “…there is no contact with the same midwife on every prenatal visit [re: continuity of care], so there is no trusting relationship between midwives and women… this whole thing makes any implementation of the educational role really difficult”*.
- *Participant 7: "…the time [a midwife] has with the woman is only 3–4 days until discharge, there is no community midwifery****.**** This is a deterrent to assuming an educational role in the first place…”*.

#### Social Opportunity

Medicalization of birth was viewed as a big contributor to the reduced opportunity for a midwife to be an educator (C). Physician dominance is sustaining power dynamics (C). This is reinforced by the healthcare system itself as well as socio-cultural norms (A, C).

- Participant 5: “…the presentation of the gynecologist as an authority”.
- Participant 7: “[Re: relationship with doctor]…this is what the system cultivates”.

Unfamiliarity or even misconceptions about the role of midwives are alienating the midwife from developing a direct and meaningful relationship with pregnant women (A, C).

- Participant 8: “**…**unsatisfactory communication between midwives and women resulting in lack of trust and unwillingness to collaborate [with the midwife]”.

This is reinforced by the lack of role clarity and referral system (A, C).

- Participant 2: “[re: lack of referral system] A midwife should be able to monitor a normal pregnancy, provide advice and refer where needed to the appropriate health professional”.
- Participant 8: "… lack of collaboration with other health professionals and links with provided services, …”

The lack of interprofessional collaboration extends to the policy level (B). The need for central coordination and closer relationships between professional associations was identified (C). Weak links between scientific bodies in the field of maternal and child health act as a barrier in developing a common policy, guidelines and standard protocols (roles, actions, referrals, etc.) (A, C).

- Participant 9 – “No common code of practice or guidelines, which if existed the problem would be resolved”

#### Physical Capability

Even though many of the identified barriers relate to organizational factors and culture (B), physical capability may also be an issue as a result of staff shortages and heavy workload (A, C). This may create conditions of cognitive overload affecting the willingness to perform the activity, which is described as ‘demanding’ if it were to be effective and personalized.

- Participant 5: *"… staff shortages is already a problem,…, if an individualized care approach is to be adopted an even larger number of midwives will be required".*

#### Psychological Capability

As an activity, it is out of direct control, especially when others things are perceived by the system as having higher priority (A, B, C). However, there might also be issues with procedural knowledge (e.g. how to perform this role) as well as “knowledge” base in general (C). At times, this might result in avoidance to engage in the role due to perceived lack of confidence (A, C).

- Participant 9: *"I noticed that each midwife offers different ‘knowledge' [re: information] to new mums, resulting in confusion and misinformation. This is done either…or … or due to actual lack of knowledge by the midwife”.*

Specific skills and competences are needed to effectively and competently perform this role and the need for skill development and assessment processes was identified (A, C).

- Participant 4: *"Better training. The training [re: continuous education] is inadequate"*
- Participant 1: “*The skills necessary for a midwife who works in the ward and operates within routine practice are different to those of the midwife who works with pregnant women [Re: antenatal clinic]”.*
- Participant 7: *“There is no form of skill assessment”*

Skills and competences that might need strengthening include: pedagogical methods, health and interpersonal communication, digital skills and use of technology, cultural competence, inter-professional collaboration, critical appraisal and evidence-based practice (C).

- Participant 11: "*Today's midwives must be well-trained, equipped with best knowledge, experience and skills. A midwife must be in a position to understand, cooperate and communicate, being able to justify every procedure and intervention based on available evidence”.*
- Participant 8: *“…adequate continuous education …the ability of midwives to combine their clinical knowledge and skills with interpersonal and transcultural skills".*

Participants identified the need for professional development to be frequent, systematic, in-service and interprofessional**,** with emphasis on experiential learning (C). They also identified the need for a specialization in Antenatal Education, currently lacking (A, C).

- Participant 1: *"There [re: Antenatal Care Clinic], it is much more likely for a midwife to be called upon to answer questions and thus in this way get the chance to develop into the educational role every day…".*
- Participant 10: *“Midwives involved in antenatal education should follow a specialization programme to become expert educators”*

#### Reflective Motivation

AE is viewed as a core function of the professional role (B) and described as a duty (A, C).

- Participant 8: "[*A midwife] has an important role, not only for the women but also for the whole family, and the community. Her work includes antenatal education and preparation for parenting but can be extended to women's health, sexual and reproductive health, and pediatric care*".
- Participant 11: *"A midwife has a duty to provide equal care to all … we need to appreciate the seriousness of the profession we have chosen”*

While intentions are strong, setting goals is difficult when other activities perceived as more necessary and urgent take priority (A, B, C). AE and the midwifery-led model of care are not valued by the current system (A, C).

- Participant 11: *“…midwives work with significant limitations in terms of their autonomy.”*
- Participant 2: *“…no emphasis on antenatal education and care”.*
- Participant 4: *"One factor that would facilitate the role of the midwife in the future is the implementation of personalized midwifery care. That is, to have a midwife for every four women, for example, so that there is time to dedicate to each woman individually"*

The opportunity afforded by newly RM to apply what they learned at University is reduced by a theory–practice gap (Participant 3: *"Theory is completely different from practice")*, making it difficult for a newcomer midwife to differentiate from predominant practices (A). The role of the “experienced midwife educator” was identified as an asset and an opportunity by providing observable role models (A, C).

- Participant 5: *"… through in-hospital mentoring, midwives [re: experienced midwives] can contribute by providing an example to follow".*
- Participant 7: *"… the informal process by which a midwife acquires the common culture of midwifery, the values, beliefs, attitudes, patterns of behavior and social identity of midwifery"*

Descriptions of the benefits of AE included a range of positive outcome expectancies (A, B, C), even though these were often described as distal goals rather than tangible outcomes, such as: personalised care, trusting relationships and cooperative alliances etc. (A). At the same time, certain aspects of the healthcare system might influence midwives’ beliefs about the effectiveness or even feasibility of AE (A, C). The lack of continuity of care and community midwifery are perceived as ‘deterrents’ (i.e. “*is it worth it?*” or “*is it even feasible?*”).

- Participant 9: *“…there is no contact with the same midwife between successive appointments [re: continuation of care]”*
- Participant 4: *"… after women leave the maternity clinic, there is no contact with the midwives… this is a problem”.*

Furthermore, physician dominance might create conditions for anticipated regret as a disagreement in opinion or confrontation may arise (C). Unclear role boundaries and weak interprofessional collaboration (B) seem to sustain power dynamics, creating situations which undermine the role of the midwife (A, C).

- Participant 7: "*A pregnant woman develops a relationship only with her doctor, she only sees the doctor, and has confidence in what he/she tells her, the doctor is the person to refer to for questions”.*
- Participant 2: *"…the medicalization of the profession but also the mentality of many Cypriots who always seek the opinion of the doctor".*

There appeared to be a ‘struggle’ between group conformity (i.e. “*accepted way things are*”, “*indifference*”, “*no initiative*”) and determination (“*courage to dare*”).

- Participant 7: "The midwife has accepted this way of working …as a result she does not train more, does not specialize, does not take initiative and does not assume an educational role beyond the moment of childbirth and for breastfeeding support”
- Participant 1: “Today's midwives must have the courage to dare to showcase their abilities in order to promote their autonomy as midwives”

Indeed, the educational role was perceived as the means to showcase the professional role and further enhance the autonomy of the profession (A, C).

- Participant 11: "… expanding the role of the midwife has the potential to provide high quality continuous maternity care to women, which increases the job satisfaction of midwives, promotes and enhances professional autonomy and responsibility in the workplace”
- Participant 4: “… and that’s why there should be Community Midwifery, giving the midwives the opportunity to promote their educational role"

#### Automatic Motivation

Emotional responses to the role were generally positive (B, C), stemming from a sense of satisfaction when successfully engaging in this role but also pride about specific achievements that showcase the midwifery model of care; for example, the recent establishment of the first midwifery-led birth centre offered optimism (C). However, the activity may also be a source of negative emotions e.g. discomfort due to unfriendly spaces, lack of trust due to contradicting advice from physicians (C). There were also negative emotions caused by the inability to provide equal care to all due to lack of time and/or linguistic barriers (A, C).

- Participant 10: “…this is without intention, this problem of unequal provision of care, is without intention, but due to the barriers that exist ".
- Participant 11: "… care should not be impersonal and each woman should feel safe and that she can trust what the midwife tells her…”

While intentions to engage in AE appear strong (B), they may not be stable but “depend on the will, desire and time” (A). Dissatisfaction with the current situation may lead to pessimism (“…any implementation of the educational role by the midwife really difficult”). Guidelines, protocols and practice standards are viewed as vital as they would reinforce procedural knowledge (“know-how”) and reduce the anticipated regret of potential differences in opinion (C). Currently, there are no incentives or rewards (C). Even opportunities provided for professional development do not seem to be equally distributed among staff (C). The civil servant status in the public sector, no distribution of roles and responsibilities based on skills and abilities and lack of evaluation for career progression are disincentives (A, C).

- Participant 8: “…better distribution of roles and responsibilities based on needs, abilities and resources"
- Participant 3: "Better evaluation system…. more opportunities to gain experience".

### Phase 2: intervention mapping

The ‘behaviour diagnosis’ suggested that the phenomenon is complex, spanning micro- and macro-organisational levels. Since no single intervention may be as effective in impacting change, the research team opted for combining rather than prioritizing intervention functions to impact Capability, Opportunity and Motivation of midwives to actively engage in AE. Thus, the mapping exercise described a feasible, yet pluralistic, intervention within the Cypriot context while the focus was on capitalizing on a newly introduced digital tool (Baby Buddy) that may facilitate the process. Figure 1 presents an overview of the seven intervention functions chosen by the research team during the workshop along with related policy categories to deliver them. A narrative account follows, while a list of eighteen possible BCTs as identified during the workshop, along with relevant justification, appear in Additional File 6.

#### Enablement and re-structuring through environmental planning

As a digital tool, Baby Buddy offers opportunities for re-thinking and re-structuring the way AE is delivered. With rich material covering the period from early pregnancy to the first six months of the baby’s life, it offers a low-cost high-quality alternative to the current lack of educational material, especially as it is available in four additional languages (English, Turkish, Arabic and Russian). Material can be used as part of formal activities (i.e. antenatal class) but, as a digital tool, it is particularly suited in assisting the educational role during routine appointments, characterized by limited time (e.g. recommending certain videos or articles). Use of Baby Buddy would allow to structure a meaningful conversation during consultation while transcending physical barriers (e.g. unsuitable spaces, privacy issues). Furthermore, Baby Buddy resources can also be used independently from the platform to affect Automatic Motivation e.g. videos playing in waiting rooms’ monitors and/or FAQs provided as conventional leaflets. This type of use may hold the potential to provide cues for parents-to-be to initiate a conversation as well as memory triggers for providers to engage in AE. To this end, maternity clinics can be further enhanced with adding objects at the point-of-care, such as Baby Buddy avatars, posters, leaflets and cards.

#### Training, modelling and incentivization through Service Provision

As the only state-funded University with a Midwifery programme, a training service could be provided to strengthen the skills and competences needed to be an ‘educator’. This should include experiential skills e.g. in-hospital practical training on how to engage with pregnant women using Baby Buddy. As attentional control is influenced by workload demands, a necessary component of training is reinforcing the self-belief that the activity can be performed and arguing against self-doubts. Furthermore, the University can affect change through its clinical training and the selection of mentors. In fact, the role of mentors was one of the key elements identified for inducing motivation and strengthening the sense of professional identity. Experienced midwives can participate in a “*Baby Buddy Champions*” train-the-trainer programme. Other than a social reward for the first trainers through peer-recognition, the programme can offer tangible incentives for other midwives completing the training through accreditation by the Cyprus Nurses and Midwives Association, a necessary requirement for renewal of registration. Baby Buddy Champions can provide “credible role models” in practice as well as on video. “Best practice” examples can feature in the Baby Buddy video library to induce a cognitive shift in providers and service users alike by providing observable examples of user-provider communication.

#### Modelling and persuasion through communication and marketing

Many of the problematic areas relate to the prevailing socio-cultural norms, mainly as a consequence of the medicalization of birth. Cultivating reflection on attitudes and beliefs around the professional role, identity and status of midwives in Cyprus may be necessary. It might be useful to suggest the deliberate adoption of a new perspective on AE and further influence positive emotions around claiming the education role as key in showcasing the autonomous role of the midwife. Drawing attention may be needed to discrepancies between current practices and self-identification as proponents of the women’s’ rights to Respectful Maternity Care. Through persuasive communication, it might also be necessary to re-frame the concept of AE from a highly structured formal activity in physical space (i.e. the “antenatal class”, currently the norm) to promoting the concept of “*making every contact count*”. Baby Buddy can be framed as a solution to some perceived barriers as well as anticipated regrets stemming from potential inter-professional group power conflicts. The latter is particularly suitable since Baby Buddy material was co-created in consultation with all national scientific bodies**.** Nevertheless, highlighting social approval might also be important through, for example, statements by professional leaders about the central role of midwives and/or testimonials promoted on Baby Buddy’s and other social media platforms about the positive experience of pregnant women with their midwife. A communication and marketing strategy using a variety of media could be directed towards midwives themselves but also the public aiming at highlighting the role of the midwife as the ‘educator’ and promoting AE as a “window of opportunity” for promoting the health and well-being of the whole family.

## Discussion

### Main findings

Midwives view AE as a core function of their role; yet it is not prioritized nor supported by current practices. The medicalization of birth is alienating midwives who nevertheless view the educational role as a means to enhance the autonomy of the profession. Enablement (e.g. Baby Buddy material) and environmental restructuring (e.g. prompts and triggers), training (e.g. skills strengthening), incentivization (e.g. continuous education accreditation) and persuasion (e.g. reflection on professional identity) were identified as promising intervention functions. Modelling may also be necessary both for providing “credible models” for the role itself as well as re-framing AE through the concept of ‘*making every contact count’*.

### Strengths and limitations

The use of both COM-B and TDF to guide data collection and analysis offered a structured approach in identifying and classifying influences on the target behaviour. An inductive or abductive content analysis approach may have produced different interpretations and new insights. In this study, data were analyzed deductively, opting for a low abstraction level and low degree of interpretation, as per the study purpose resulting in a high degree of dependability [36]. The complimentary nature of the three studies allowed method and informant triangulation (in-training and registered midwives), further supporting the credibility. All participants had experience of the behaviour under study and wanted to share the challenges. It is interesting, however, that often participants did not refer to their own experience but to ‘others’ or what ‘some midwives’ do, possibly as a reflection of practice through the eyes of peers.

While data saturation was reached (also see relevant point in Additional File 2), it is unclear whether theoretical saturation was achieved due to the purposive sample of the focus group and voluntary participation in the workshop. Participants may represent a motivated group while a more heterogeneous sample may have identified additional barriers and/or facilitators. Future studies should assess the replicability of the findings and evaluate the appropriateness of the identified interventions directly with the target group and stakeholders. A strength in the present study is that study participants had the opportunity to verify the recognizability of the experience and reasonableness of presentation. In fact, the NGT classification was presented during a plenary session to all delegates at the launch event, confirming resonance and cultivating a raised consciousness among the group. In the context of designing the intervention, the findings were presented to the wider project team, who identified the readability of the narrative, reasonableness of interpretation and more importantly the relevance of the representation in informing the selection of intervention functions and techniques.

Regarding the quantitative survey, even though the sample is small, and did not allow a formal validation of the measurement scale or an exploration of differences according to socio-demographic, work-related characteristics and setting (public vs private sector), it allowed piloting the questionnaire before the launch of a larger paper-form survey which is currently on-going across all maternity clinics. This will provide the opportunity to assess the consistency of the findings with a representative sample of midwives. Finally, as the findings also indicate, midwives’ practices are influenced by the behaviour of other actors (other providers and pregnant women) and organizational factors (clinic policies and leadership). Thus, future studies could approach the issue as a complex adaptive system, especially as many of the identified intervention components concern the micro- and macro-organizational level beyond the direct control or influence of the research team.

### Barriers and facilitators related to midwives’ educational role

Barriers to midwives’ educational role are likely to be topic- (e.g. smoking cessation versus mental health screening), healthcare system- (e.g. status of midwifery) and context- (e.g. low versus high-resource setting) specific, thus, making comparison of findings across international settings difficult. The rapid review (preparatory phase) identified fifteen studies published in 2012–2020, originating from a small number of settings; five from the UK, six from Australia and four from New Zealand, Canada, Tanzania and Uganda (see Additional file 5). Seven used predominantly qualitative methods to collect information, such as focus groups, interviews or written responses [37–44]. Four employed a paper-based or online questionnaire survey with varying number of items (*N* = 40–56) [45–48] and three mixed-methods [28, 49, 50]. Qualitative and mixed-method studies employed the COM-B [38, 39, 49, 50] or the TDF [28, 37, 40–44] to inform the interview guide and code the material.

The majority of studies do not address the education and/or health promotion role of midwives in general but focus on exploring barriers to implementing a specific policy, programme or intervention; for example, smoking cessation support [40, 45, 48], advice for physical activity [38], alcohol [46] or nutrition [37], place of birth discussions [49], Down syndrome prenatal screening [42], antenatal Corticosteroid Clinical Practice guidelines [43] and antenatal magnesium sulphate for fetal neuroprotection guidelines [44]. A number of studies focus on special population groups, such as obese pregnant women [47], indigenous women [50] and women with refugee background [41].

The study by McLellan et al. (2019) is more similar to the current study in terms of aims [28]. Focusing on multiple health promotion practice behaviours (termed, HePPBes), the authors performed semi-structured interviews with eleven community midwives, followed by an online survey. Similar to our findings, the authors identified that UK midwives hold strong beliefs about the importance of health promotion and their own role. Clinical work overload, lack of continuity of care, quality of the relationship with pregnant women and lack of training also emerged as some of the main barriers, even though, unlike Cyprus, the role of the midwife as a Public Health Practitioner is institutionalized in the UK.

Overall, lack of time, competing priorities and/or insufficient resources (Physical Opportunity: Context and Resources) in parallel with weak procedural or specialized knowledge and insufficient practical skills training opportunities (Psychological Capability: Knowledge and Skills) are factors which consistently emerge across studies irrespective of whether they originate from a higher- or lower-resource setting. Thus, strengthening skill training is a common suggestion across studies, as is enablement and environmental planning with providing the necessary resources, in the form of material and tools to facilitate the task (e.g. information leaflets, check lists), increase habit or performance (e.g. audits and feedback) and/or provide prompts and cues to deal with attentional control.

However, as this study found, lack of resources is only one of several barriers in the highly medicalized maternity care services in Cyprus. The prevalence of over-medicalization is prominently reflected in the highest caesarean section rate in the EU (approaching 60%), coupled with very high episiotomy rates (over 70% of all vaginal births according to most recent official Perinatal Health statistics). In this environment, the autonomous role, scope of practice and professional recognition of midwives is significantly restricted by the system and socio-cultural norms pertaining to users and providers.

A qualitative study exploring the perceptions of midwives in Cyprus as advocates of normal childbirth has, characteristically, concluded that “In Cyprus, midwifery in dying” against a continuing climate of ‘physician dominance’ [51]. The study identified ‘lack of institutional support’, and this was before the recent restructuring of the General Healthcare System (GeSY). While the 2020 reforms could have paved the path to a more autonomous role for the Cyprus midwife, it has instead retained, if not exacerbated, previous distortions, which limit the scope of midwifery care. It appears that essential aspects of care are undervalued by the system, which undermines the acceptance of the midwifery model of care among the general public. Interestingly, a qualitative study among midwives in Germany suggested that, even in a less medicalised system by comparison, a more prominent presence of midwifery care in the general public, in combination with adequate remuneration of midwifery services, are needed to counteract the increasing medicalisation of birth [52].

While Cyprus is a prime example where current socio-political arrangements are generally unsupportive, this study also identified issues with procedural knowledge and skills *(“how to do it”*). Lack of these skills may result in perceived lack of confidence and avoidance, especially in the absence of a standard code of practice. Indeed, a long line of research suggests that healthcare providers may not use opportunities consistently and/or effectively during their routine interactions with service-users to “*make every contact count*” [53]. However, ‘Making Every Contact Count’ (MECC) has not been translated in a consistent approach to training, with paucity of research evidence to support the impact of MECC training on improved professional practices [54]. A recent study from the UK evaluated the acceptability and feasibility of “Healthy Conversation Skills”, a brief interpersonal communication intervention, as part of a larger randomized control trial, to strengthen health-promoting behaviour change in routine maternity care [55]. While this increased consultation times, the study found that women valued the experience, especially since the focus was on their own health as well as their baby. Midwives reported that they developed skills and confidence over time but identified that appropriate training should be incorporated into midwifery education and continuous professional development. Even though there is lack of good quality studies on the impact of different skills training among nurses and midwives [56, 57], there is some evidence that experiential practice through real-life situations (simulations and/or peer role-play) are more effective in cultivating these skills. However, how it should be done (taught at University) is not necessarily aligned with what is practiced (in hospitals) and hence experienced in clinical settings (by students). The study findings suggest that this theory–practice gap may be reproducing sub-optimal practices, making it especially difficult for a newcomer midwife to differentiate from predominant practices. As Reynolds et al. (2020) discuss in a position paper, skilled clinical supervisors and mentors, who are competent educators themselves, are needed who can both opportunistically as well as proactively plan “teaching moment” opportunities to enhance the work-place learning for nurses and midwifery students [58].

### Implications for future research, practice and policy

The phenomenon is complex, and pertains to long-standing structural barriers. No single intervention may be effective in impacting change; however, embedding Baby Buddy in clinical practice may be used as a vehicle to facilitate some aspects of the process. While digital tools, such as Baby Buddy Cyprus, have the potential to transform AE, effective and motivated antenatal educators are needed in the first place in order to successfully embed digital tools in clinical and community practice. Baby Buddy can assist in the performance of the educational role by increasing the means (i.e. lack of educational material) and reducing barriers (e.g. physical and linguistic), thus impacting *Capability* and *Opportunity* to perform the role in a suboptimal setting and context. However, future research and action in the first instance should be directed towards improving the necessary skills to competently perform this role.

A 2018 Cochrane review identified 87 implementation interventions for strengthening user-provider interactions in the context of shared-decision making [59], of which only one concerned Maternity Care services [60]. A recently published secondary analysis of the above review employed the COM-B approach to identify the most effective combination of intervention functions and specific BCTs [61]. The overall conclusion, even though not specific to Maternity Care services, was that (a) the most commonly used single function was Educational-based interventions (73 out of 87 studies) and the most commonly used BCT was “Instruction on how to perform the behaviour” (43 studies). However, the review identified that interventions were more likely to be effective if several functions, beyond Education and Training, were incorporated, such as Modelling and Enablement.

The proposed intervention in this study was shaped on the basis of expert panel consensus. This will need to be refined and adapted based on an assessment of appropriateness and feasibility among the actual target group. Other than some notable exceptions, very few studies incorporate an explicit process of intervention mapping involving the target group. Henshall et al. (2018) engaged with midwives in co-creation workshops to evaluate potential interventions to strengthen the quality and content of place of birth discussions [49]. Similarly, Campbell et al. (2017) engaged in a participatory process in order to select promising interventions to improve postpartum diabetes screening among Indigenous women with gestational diabetes [50]. Before the implementation of any intervention, the various identified components need to refined, prioritized and adapted accordingly, adopting a participatory process directly involving the actual target group and stakeholders. In addition to an assessment of appropriateness and feasibility along the APEASE criteria, the process needs to consider the study aims and design, material, outcomes and related measures to document change.

An initial assessment of the various intervention components according to the APEASE criteria identified a number of issues and possible unintended consequences in the context of scalability, sustainability and acceptability. For instance, affecting a cognitive shift in the role of midwives would be limited if this role is not institutionally supported, especially in the private sector which represents over 70% of births in Cyprus but differing levels of autonomy create differential conditions of applicability. Efforts should also concentrate on developing guidelines and protocols in collaboration with professional associations through a similar academia-led participatory action process used to shape the content of Baby Buddy. Furthermore, advocacy efforts are needed towards strengthening the professional role of midwives, for instance (a) enforcement of current legislation on minimum staff requirements in maternity clinics and (b) improved direct access to midwives in terms of number of visits (currently only six, only after referral by Obstetrician-Gynecologist) and range of services reimbursed by the newly established General Healthcare System (GeSY).

## Conclusions

Barriers to being an effective antenatal educator are several, originating from an unsupportive system and wider socio-cultural norms pertaining to users and providers. Through a theory-driven research-informed process, the study identified various components necessary to impact change through better-prepared, better-equipped and better-received midwives.

## Supplementary Information

**Additional file 1. **Definitions of the 6 COM-B model components and relationship with the 14 domains of the Theoretical Domains Framework (TDF).

**Additional file 2. **Reporting standards (COREQ and StaRI) as well as transparency criteria for replicability.

**Additional file 3. **Open-ended question and semi-structured interview guide for the focus group session.

**Additional file 4. **Survey questionnaire referring to A=Action, C=context, T=time, and Ta=target with equivalent Greek translation of items referring to antenatal education and counseling [A] during routine antenatal appointments [C, T] with pregnant women and partners [Ta].

**Additional file 5. **Rapid Literature Review: Barriers and enablers to midwives’ educational role as depicted in qualitative and/or quantitative studies that used the COM-B and/or the Theoretical Domains Framework.

**Additional file 6. **Behavioural Change Techniques (BCTs) to support the identified intervention functions.

## Data Availability

All data generated or analysed during this study are included in this published article and its supplementary information files and/or are available from the corresponding author on reasonable request.

## Abbreviations

AE: Antenatal education
BCTs: Behaviour Change Techniques
BCW: Behaviour Change Wheel
COM-B: Capability, Opportunity, Motivation-Behaviour
COREQ: COnsolidated criteria for REporting Qualitative studies
GeSY: General Health System [in Greek]
HP: Healthcare providers
PAR: Participatory Action Research
RM: Registered Midwife/ves
StaRI: Standards for Reporting Implementation studies
TDF: Theoretical Domains Framework
WHO: World Health Organization

## References

[1] Brady V, Lalor J (2017). Space for human connection in antenatal education: Uncovering women's hopes using Participatory Action Research. Midwifery.

[2] Spiteri G, Borg Xuereb R, Carrick-Sen D, Kaner E, Martin CR (2014). Preparation for parenthood: a concept analysis. J Reprod Infant Psychol.

[3] Stylianides K, Middleton N, Kouta C, Raftopoulos V (2016). The Role of Emotional Intelligence and Postpartum Depression in Predicting Mothers’ Satisfaction with Quality of Co-Operation with Obstetricians and Midwives. Int J Caring Sci.

[4] Economou M, Kolokotroni O, Paphiti-Demetriou I, Kouta C, Lambrinou E, Hadjigeorgiou E, Hadjiona V, Tryfonos F, Philippou E, Middleton N (2018). Prevalence of breast-feeding and exclusive breast-feeding at 48 h after birth and up to the sixth month in Cyprus: the BrEaST start in life project. Public Health Nutr.

[5] Novick G (2009). Women's experience of prenatal care: an integrative review. J Midwifery Womens Health.

[6] Nolan ML (2009). Information giving and education in pregnancy: a review of qualitative studies. J Perinat Educ.

[7] Entsieh AA, Hallström IK (2016). First-time parents’ prenatal needs for early *parenthood* preparation-A systematic review and meta-synthesis of qualitative literature. Midwifery.

[8] Downe S, Finlayson K, Tunçalp Ӧ, Metin Gülmezoglu A (2016). What matters to women: a systematic scoping review to identify the processes and outcomes of antenatal care provision that are important to healthy pregnant women. BJOG.

[9] Rowe RE, Garcia JO (2003). Social class, ethnicity and attendance for antenatal care in the United Kingdom: a systematic review. J Public Health.

[10] Balaam MC, Akerjordet K, Lyberg A, Kaiser B, Schoening E, Fredriksen AM, Ensel A, Gouni O, Severinsson E (2013). A qualitative review of migrant women's perceptions of their needs and experiences related to pregnancy and childbirth. J Adv Nurs.

[11] Baron R, Manniën J, te Velde SJ, Klomp T, Hutton EK, Brug J (2015). Socio-demographic inequalities across a range of health status indicators and health behaviours among pregnant women in prenatal primary care: a cross-sectional study. BMC Pregnancy Childbirth.

[12] Atkinson L, Shaw RL, French DP (2016). Is pregnancy a teachable moment for diet and physical activity behaviour change? An interpretative phenomenological analysis of the experiences of women during their first pregnancy. Br J Health Psychol.

[13] Fry-Bowers EK, Maliski S, Lewis MA, Macabasco-O'Connell A, DiMatteo R (2014). The association of health literacy, social support, self-efficacy and interpersonal interactions with health care providers in low-income Latina mothers. J Pediatr Nurs.

[14] Kozhimannil KB, Attanasio LB, Yang YT, Avery MD, Declercq E (2015). Midwifery care and patient–provider communication in maternity decisions in the United States. Matern Child Health J.

[15] World Health Organization. WHO recommendations on antenatal care for a positive pregnancy experience. World Health Organization; 2016. https://www.who.int/publications/i/item/9789241549912. Accessed 01 Oct 2022. 28079998

[16] Arrish J, Yeatman H, Williamson M (2017). Midwives' role in providing nutrition advice during pregnancy: Meeting the challenges? A qualitative study. Nutr Res Pract.

[17] Holton S, East C, Fisher J (2017). Weight management during pregnancy: a qualitative study of women's and care providers' experiences and perspectives. BMC Pregnancy Childbirth.

[18] McCann MT, Newson L, Burden C, Rooney JS, Charnley MS, Abayomi JC (2018). A qualitative study exploring midwives' perceptions and knowledge of maternal obesity: reflecting on their experiences of providing healthy eating and weight management advice to pregnant women. Matern Child Nutr.

[19] Tripp N, Hainey K, Liu A, Poulton A, Peek M, Kim J, Nanan R (2014). An emerging model of maternity care: smartphone, midwife, doctor?. Women Birth.

[20] Yvonne FM (2010). Doing mixed methods research pragmatically: Implications for the rediscovery of pragmatism as a research paradigm. J Mixed Methods Res.

[21] Kelly LM, Cordeiro M (2020). Three principles of pragmatism for research on organizational processes. Methodological Innovations.

[22] Michie S, Atkins L, West R (2014). The behaviour change wheel. A guide to designing interventions.

[23] Cane J, O’Connor D, Michie S (2012). Validation of the theoretical domains framework for use in behaviour change and implementation research. Implement Sci.

[24] Michie, S., Wood, C.E., Johnston, M., Abraham, C., Francis, J. and Hardeman, W. Behaviour change techniques: the development and evaluation of a taxonomic method for reporting and describing behaviour change interventions (a suite of five studies involving consensus methods, randomised controlled trials and analysis of qualitative data). Health Technology Assessment. 2015;19(99). 10.3310/hta19990. 10.3310/hta19990 PMC4781650 26616119

[25] Aguinis H, Solarino AM (2019). Transparency and replicability in qualitative research: the case of interviews with elite informants. Strateg Manag J.

[26] Alexander KE, Brijnath B, Mazza D (2014). Barriers and enablers to delivery of the Healthy Kids Check: an analysis informed by the Theoretical Domains Framework and COM-B model. Implement Sci.

[27] Atkins L, Hunkeler EM, Jensen CD, Michie S, Lee JK, Doubeni CA, Zauber AG, Levin TR, Quinn VP, Corley DA (2016). Factors influencing variation in physician adenoma detection rates: a theory-based approach for performance improvement. Gastrointest Endosc.

[28] McLellan JM, O’Carroll RE, Vheyne H, Dombrowski SU (2019). Investigating Midwives’ barriers and facilitators to multiple health promotion practice behaviour: a qualitative study using the theoretical domains framework. Implement Sci.

[29] Gale NK, Heath G, Cameron E, Rashid S, Redwood S (2013). Using the framework method for the analysis of qualitative data in multi-disciplinary health research. BMC Med Res Methodol.

[30] Goldsmith LJ (2021). Using Framework Analysis in Applied Qualitative Research. Qualitative Report.

[31] Ramanadhan S, Revette AC, Lee RM, Aveling EL. Pragmatic approaches to analyzing qualitative data for implementation science: an introduction. Implementation Sci Commun. 2021; 70(2). 10.1186/s43058-021-00174-1. 10.1186/s43058-021-00174-1 PMC8243847 34187595

[32] Huijg JM, Gebhardt WA, Crone MR, Dusseldorp E, Presseau J (2014). Discriminant content validity of a theoretical domains framework questionnaire for use in implementation research. Implement Sci.

[33] Huijg JM, Gebhardt WA, Dusseldorp E, Verheijden MW, van der Zouwe N, Middelkoop BJ, Crone MR (2014). Measuring determinants of implementation behavior: psychometric properties of a questionnaire based on the theoretical domains framework. Implement Sci.

[34] Atkins L, Francis J, Islam R, O’Connor D, Patey A, Ivers N, Foy R, Duncan EM, Colquhoun H, Grimshaw JM (2017). A guide to using the theoretical domains framework of behaviour change to investigate implementation problems. Implement Sci.

[35] Bengtsson M (2016). How to plan and perform a qualitative study using content analysis. NursingPlus Open.

[36] Graneheim UH, Lindgren BM, Lundman B (2017). Methodological challenges in qualitative content analysis: a discussion paper. Nurse Educ Today.

[37] Saronga N, Burrows T, Collins CE, Mosha IH, Sunguya BF, Rollo ME (2020). Nutrition services offered to pregnant women attending antenatal clinics in Dar es Salaam, Tanzania: a qualitative study. Midwifery.

[38] Lucas G, Olander EK, Salmon D (2020). Healthcare professionals’ views on supporting young mothers with eating and moving during and after pregnancy: an interview study using the COM-B framework. Health Soc Care Community.

[39] Wakida EK, Obua C, Rukundo GZ, Maling S, Talib ZM, Okello ES (2018). Barriers and facilitators to the integration of mental health services into primary healthcare: a qualitative study among Ugandan primary care providers using the COM-B framework. BMC Health Serv Res.

[40] Longman JM, Adams CM, Johnston JJ, Passey ME (2018). Improving implementation of the smoking cessation guidelines with pregnant women: How to support clinicians?. Midwifery.

[41] Nithianandan N, Gibson-Helm M, McBride J, Binny A, Gray KM, East C, Boyle JA (2016). Factors affecting implementation of perinatal mental health screening in women of refugee background. Implement Sci.

[42] Lépine J, Portocarrero ME, Delanoë A, Robitaille H, Lévesque I, Rousseau F, Wilson BJ, Giguère AM, Légaré F (2016). What factors influence health professionals to use decision aids for Down syndrome prenatal screening?. BMC Pregnancy Childbirth.

[43] Mc Goldrick EL, Crawford T, Brown JA, Groom KM, Crowther CA (2016). Identifying the barriers and enablers in the implementation of the New Zealand and Australian Antenatal Corticosteroid Clinical Practice Guidelines. BMC Health Serv Res.

[44] Bain E, Bubner T, Ashwood P, Van Ryswyk E, Simmonds L, Reid S, Middleton P, Crowther CA (2015). Barriers and enablers to implementing antenatal magnesium sulphate for fetal neuroprotection guidelines: a study using the theoretical domains framework. BMC Pregnancy Childbirth.

[45] Passey ME, Longman JM, Adams C, Johnston JJ, Simms J, Rolfe M (2020). Factors associated with provision of smoking cessation support to pregnant women–a cross-sectional survey of midwives in New South Wales, Australia. BMC Pregnancy Childbirth.

[46] Doherty E, Kingsland M, Wiggers J, Anderson AE, Elliott EJ, Symonds I, Tully B, Dray J, Wolfenden L (2020). Barriers to the implementation of clinical guidelines for maternal alcohol consumption in antenatal services: a survey using the theoretical domains framework. Health Promot J Austr.

[47] McParlin C, Bell R, Robson SC, Muirhead CR, Araújo-Soares V (2017). What helps or hinders midwives to implement physical activity guidelines for obese pregnant women? A questionnaire survey using the Theoretical Domains Framework. Midwifery.

[48] Beenstock J, Sniehotta FF, White M, Bell R, Milne EM, Araujo-Soares V (2012). What helps and hinders midwives in engaging with pregnant women about stopping smoking? A cross-sectional survey of perceived implementation difficulties among midwives in the North East of England. Implement Sci.

[49] Henshall C, Taylor B, Goodwin L, Farre A, Jones ME, Kenyon S (2018). Improving the quality and content of midwives’ discussions with low-risk women about their options for place of birth: Co-production and evaluation of an intervention package. Midwifery.

[50] Campbell S, Roux N, Preece C, Rafter E, Davis B, Mein J, Boyle J, Fredericks B, Chamberlain C (2017). Paths to improving care of Australian Aboriginal and Torres Strait Islander women following gestational diabetes. Prim Health Care Res Dev.

[51] Hadjigeorgiou E, Coxon K (2014). In Cyprus, ‘midwifery is dying…’. A qualitative exploration of midwives' perceptions of their role as advocates for normal childbirth. Midwifery.

[52] Lohmann S, Mattern E, Ayerle GM (2018). Midwives' perceptions of women's preferences related to midwifery care in Germany: a focus group study. Midwifery.

[53] Public Health England, NHS England and Health Education England. Making Every Contact Count (MECC): Consensus statement. Public Health England. 2016. https://assets.publishing.service.gov.uk/government/uploads/system/uploads/attachment_data/file/769486/Making_Every_Contact_Count_Consensus_Statement.pdf. Accessed 01 Oct 2022.

[54] Chisholm A, Byrne-Davis L, Peters S, Beenstock J, Gilman S, Hart J (2020). Online behaviour change technique training to support healthcare staff ‘Make Every Contact Count’. BMC Health Serv Res.

[55] Lawrence W, Vogel C, Strömmer S, Morris T, Treadgold B, Watson D, Hart K, McGill K, Hammond J, Harvey NC, Cooper C (2020). How can we best use opportunities provided by routine maternity care to engage women in improving their diets and health?. Matern Child Nutr.

[56] MacDonald-Wicks L, Levett-Jones T (2012). Effective teaching of communication to health professional undergraduate and postgraduate students: a systematic review. JBI Evid Synth.

[57] Moore PM, Mercado SR, Artigues MG, Lawrie TA (2013). Communication skills training for healthcare professionals working with people who have cancer. Cochrane Database Syst Rev.

[58] Reynolds LM, Attenborough J, Halse J (2020). Nurses as educators: creating teachable moments in practice. Nurs Times.

[59] Légaré F, Adekpedjou R, Stacey D, Turcotte S, Kryworuchko J, Graham ID, Lyddiatt A, Politi MC, Thomson R, Elwyn G, Donner-Banzhoff N (2018). Interventions for increasing the use of shared decision making by healthcare professionals. Cochrane Database Syst Rev.

[60] O'Cathain A, Walters SJ, Nicholl JP, Thomas KJ, Kirkham M (2002). Use of evidence based leaflets to promote informed choice in maternity care: randomised controlled trial in everyday practice. BMJ.

[61] Agbadjé TT, Elidor H, Perin MS, Adekpedjou R, Légaré F (2020). Towards a taxonomy of behavior change techniques for promoting shared decision making. Implement Sci.

