# Supplementary material for: Identifying barriers to the educational role of midwives in Cyprus and defining determinants in behaviour terms using the Behaviour Change Wheel: a mixed-method formative study
Source: BMC Health Serv Res. 2022 Oct 5;22:1233. doi: 10.1186/s12913-022-08599-7 (PMC9534462; doi:10.1186/s12913-022-08599-7)
Supplement: Supplementary file 1 — Additional file 1. Definitions of the 6 COM-B model components and relationship with the 14 domains of the Theoretical Domains Framework (TDF). [file 12913_2022_8599_MOESM1_ESM.pdf]

Additional File 1

Definitions of the 6 COM-B model components and relationship with the 14 domains of the Theoretical Domains Framework (TDF).

**Theoretical Domain Framework**	**COM-B**
**Domain definition**	**Component definition**
**(1) Skills (physical)**	**Physical Capability**<br>Necessary physical skill, strength or stamina to perform behaviour
**Skills (cognitive and interpersonal)**<br>Ability or proficiency acquired through practice	**Psychological Capability**<br>Knowledge and/or psychological skills, strength or stamina to engage in necessary mental processes to perform behaviour
**(2) Knowledge**<br>Awareness of something, including scientific rationale and procedural knowledge	
**(3) Memory, attention and decision process**<br>Ability to retain information, focus selectively (e.g. attention control) and choose between two or more alternatives (e.g. decision making), which might also be affected by overload and tiredness	
**(4) Behavioural regulation**<br>Managing (e.g. goal setting) or changing (e.g. breaking habit) objectively observed or measured actions (e.g. self-monitoring)	
**(5) Environmental context and Resources**<br>Circumstances and context in the external environment that encourage or discourage development of skills and abilities, competence and adaptive behaviour, including available resources, physical barriers or facilitators, environmental stressors and person-environment interactions.	**Physical Opportunity**<br>Opportunity afforded by the environment involving time, resources, locations, cues, etc
**(6) Social influences**<br>Interpersonal process that can affect individuals' behaviour (e.g. socio-cultural norms, intergroup conflict) or cause individuals to change their thoughts, feelings or behaviours, including process of social comparisons, credible models and peer influences.	**Social Opportunity**<br>Opportunity afforded by interpersonal influences, social cues, cultural norms or other influences from social environment

**(7) Social and/or Professional Role and Identity**<br>Coherent set of behaviours and displayed personal qualities of an individual in a social or work setting, including professional role, role boundaries, group identity and leadership	**Reflective Motivation**<br>Reflective processes involving plans (e.g. self-conscious intentions) and evaluations (e.g. beliefs about what is good and bad)
**(8) Beliefs about Capabilities**<br>Belief in an ability, talent, or facility (e.g. perceived professional and self-competence) that an individual can put or can be empowered to put to constructive use (e.g. self-efficacy)	
**(9) Beliefs about Consequences**<br>Beliefs about the outcomes of a behaviour in a given situation which may include positive outcomes expectancies and/or anticipated regrets and consequences	
**(10) Optimism**<br>Confidence that things will happen for the best (e.g. optimism vs pessimism) or that desired goals will be attained (e.g. realistic vs unrealistic) while dealing with uncertainty	
**(11) Intentions**<br>Conscious decision to perform a behaviour (e.g. stability) or resolve to act in a certain way (e.g. determination)	
**(12) Goals**<br>Mental representations of outcomes or end states (e.g. proximal or distal goals) that an individual want to achieve (e.g. action planning and implementation intention)	
**(13) Reinforcement**<br>Increasing the probability of a response through a dependent relationship or contingency between response and given stimulus e.g. incentives, proximal or distal rewards and/or sanctions	**Automatic Motivation**<br>Automatic processes involving emotional reactions, desires, wants and needs, impulses, inhibitions, drive states and reflex responses
**(14) Emotion**<br>Complex reaction pattern by which the individual attempts to deal with a personally significant matter or event with positive (e.g. professional satisfaction) or negative feelings (e.g. stress or burn-out)	

Adapted from: Cane J, O'Connor D, Michie S. Validation of the theoretical domains framework for use in behaviour change and implementation research. Implementation science. 2012; 7(1):1-7.
