# Supplementary material for: Identifying barriers to the educational role of midwives in Cyprus and defining determinants in behaviour terms using the Behaviour Change Wheel: a mixed-method formative study
Source: BMC Health Serv Res. 2022 Oct 5;22:1233. doi: 10.1186/s12913-022-08599-7 (PMC9534462; doi:10.1186/s12913-022-08599-7)
Supplement: Supplementary file 2 — Additional file 2. Reporting standards (COREQ and StaRI) as well as transparency criteria for replicability. [file 12913_2022_8599_MOESM2_ESM.pdf]

Additional File 2
Consolidated criteria for reporting qualitative studies (COREQ): 32-item checklist

Developed from: Tong A, Sainsbury P, Craig J. Consolidated criteria for reporting qualitative research (COREQ): a 32-item checklist for interviews and focus groups. International journal for quality in health care. 2007; 19(6):349-57.

**No. Item**	**Guide questions/description**	**Reported on Page**
**Domain 1: Research team and reflexivity**		
Personal Characteristics		
1. Interviewer/facilitator	Which author/s conducted the interview or focus group?	Pg 13-14, 17 (Methods) + Pg 45-46 (Authors' contribution)
2. Credentials	What were the researcher's credentials? E.g. PhD, MD	Pg 13-14, 17 (Methods) + Pg 45-46 (Authors' contribution)
3. Occupation	What was their occupation at the time of the study?	Pg 13-14, 17 (Methods) + Pg 45-46 (Authors' contribution)
4. Gender	Was the researcher male or female?	
5. Experience and training	What experience or training did the researcher have?	Pg 13-14, 17 (Methods) + Pg 45-46 (Authors' contribution)
Relationship with participants		
6. Relationship established	Was a relationship established prior to study commencement?	Pg 13-14, 17 (Methods) + Additional file 2 (Transparency criteria)
7. Participant knowledge of the interviewer	What did the participants know about the researcher? e.g. personal goals, reasons for doing the research	Pg 13-14, 17 (Methods) + Additional file 2 (Transparency criteria)
8. Interviewer characteristics	What characteristics were reported about the interviewer/facilitator? e.g. Bias, assumptions, reasons and interests in the research topic	Pg 8 (Background), Pgs 13-14, 17 (Methods) + Pgs 45-46 (Authors' contribution) + Pgs 46-47 (Acknowledgements)
**Domain 2: study design**		
Theoretical framework		
9. Methodological orientation and Theory	What methodological orientation was stated to underpin the study? e.g. grounded theory, discourse analysis, ethnography, phenomenology, content analysis	Pg 9 (Aim) + Pgs 9-10 (Methods/Study Design)

Participant selection		
10. Sampling	How were participants selected? e.g. purposive, convenience, consecutive, snowball	Pgs 13-14 (Methods/Study A) + Pgs.16-17 (Methods/Study C) + Pg 34-35 (Strengths and Limitations)
11. Method of approach	How were participants approached? e.g. face-to-face, telephone, mail, email	Pgs 13-14 (Methods/Study A) + Pgs.16-17 (Methods/Study C)
12. Sample size	How many participants were in the study?	Pgs 13-14 (Methods/Study A) + Pgs.16-17 (Methods/Study C)
13. Non-participation	How many people refused to participate or dropped out? Reasons?	N/A
Setting		
14. Setting of data collection	Where was the data collected? e.g. home, clinic, workplace	Pgs 13-14 (Methods/Study A) + Pgs.16-17 (Methods/Study c)
15. Presence of non-participants	Was anyone else present besides the participants and researchers?	N/A
16. Description of sample	What are the important characteristics of the sample? e.g. demographic data, date	Pgs 13-14 (Methods/Study A) + Pgs.16-17 (Methods/Study C) + Pg 34-35 (Strengths and Limitations)
Data collection		
17. Interview guide	Were questions, prompts, guides provided by the authors? Was it pilot tested?	Pgs 13-14 (Methods/Study A) + Pgs.16-17 (Methods/Study C) + Additional file 3
18. Repeat interviews	Were repeat interviews carried out? If yes, how many?	Pgs 13-14 (Methods/Study A) + Pgs.16-17 (Methods/Study C) + Pg 34-35 (Strengths and Limitations)
19. Audio/visual recording	Did the research use audio or visual recording to collect the data?	Pgs 13-14 (Methods/Study A)
20. Field notes	Were field notes made during and/or after the interview or focus group?	N/A
21. Duration	What was the duration of the interviews or focus group?	Pgs 13-14 (Methods/Study A) + Pgs.16-17 (Methods/Study C)
22. Data saturation	Was data saturation discussed?	Pg 34-35 (Strengths and Limitations) + Additional file 2 (Transparency criteria)
23. Transcripts returned	Were transcripts returned to participants for comment and/or correction?	Pgs 13-14 (Methods/Study A) + Pgs.16-17 (Methods/Study C) + Pg 34-35 (Strengths and Limitations)

**Domain 3: analysis and findings**		
Data analysis		
24. Number of data coders	How many data coders coded the data?	Pgs 13-14 (Methods/Study A) + Pgs.16-17 (Methods/Study C)
25. Description of the coding tree	Did authors provide a description of the coding tree?	Pg 11 (COM-B/TDF), Pg 14 (Methods) and Additional File 1
26. Derivation of themes	Were themes identified in advance or derived from the data?	Pg 11 (COM-B/TDF), Pg 14 (Methods) and Additional File 1
27. Software	What software, if applicable, was used to manage the data?	N/A
28. Participant checking	Did participants provide feedback on the findings?	Pgs 13-14 (Methods/Study A) + Pgs.16-17 (Methods/Study C) + Pg 34-35 (Strengths and Limitations)
Reporting		
29. Quotations presented	Were participant quotations presented to illustrate the themes/findings? Was each quotation identified? e.g. participant number	Pgs 22-29 (Results) + Table 1
30. Data and findings consistent	Was there consistency between the data presented and the findings?	Pgs 19-21 (Results)
31. Clarity of major themes	Were major themes clearly presented in the findings?	Pgs 22-29 (Results) + Tables 1 and 3
32. Clarity of minor themes	Is there a description of diverse cases or discussion of minor themes?	Pg 19-21 (Results)

Standards for Reporting Implementation Studies: the StaRI checklist for completion

The StaRI standard should be referenced as: Pinnock H, Barwick M, Carpenter C, Eldridge S, Grandes G, Griffiths CJ, Rycroft-Malone J, Meissner P, Murray E, Patel A, Sheikh A, Taylor SJC for the StaRI Group. Standards for Reporting Implementation Studies (StaRI) statement. *BMJ* 2017;356:i6795

The detailed Explanation and Elaboration document, which provides the rationale and exemplar text for all these items is: Pinnock H, Barwick M, Carpenter C, Eldridge S, Grandes G, Griffiths C, Rycroft-Malone J, Meissner P, Murray E, Patel A, Sheikh A, Taylor S, for the StaRI group. Standards for Reporting Implementation Studies (StaRI). Explanation and Elaboration document. *BMJ Open* 2017 2017;7:e013318

Notes: A key concept of the StaRI standards is the dual strands of describing, on the one hand, the implementation strategy and, on the other, the clinical, healthcare, or public health intervention that is being implemented. These strands are represented as two columns in the checklist.

The primary focus of implementation science is the implementation strategy (column 1) and the expectation is that this will always be completed.

The evidence about the impact of the intervention on the targeted population should always be considered (column 2) and either health outcomes reported or robust evidence cited to support a known beneficial effect of the intervention on the health of individuals or populations.

The StaRI standards refers to the broad range of study designs employed in implementation science. Authors should refer to other reporting standards for advice on reporting specific methodological features. Conversely, whilst all items are worthy of consideration, not all items will be applicable to, or feasible within every study.

Checklist item		Reported on page #	Implementation Strategy	Reported on page #	Intervention
			"Implementation strategy" refers to how the intervention was implemented		"Intervention" refers to the healthcare or public health intervention that is being implemented.
**Title and abstract**					
Title	**1**	Pg 1	Identification as an implementation study, and description of the methodology in the title and/or keywords		
Abstract	**2**	Pgs 3-4	Identification as an implementation study, including a description of the implementation strategy to be tested, the evidence-based intervention being implemented, and defining the key implementation and health outcomes.		
**Introduction**					
Introduction	**3**	Pgs 7-8	Description of the problem, challenge or deficiency in healthcare or public health that the intervention being implemented aims to address.		
Rationale	**4**	Pgs 10-11	The scientific background and rationale for the implementation strategy (including any underpinning theory/framework/model, how it is expected to achieve its effects and any pilot work).	Additional file 1<br>Additional file 5	The scientific background and rationale for the intervention being implemented (including evidence about its effectiveness and how it is expected to achieve its effects).

Aims and objectives	**5**	Pgs 8-9	The aims of the study, differentiating between implementation objectives and any intervention objectives.		
**Methods: description**					
Design	**6**	Pgs 9-10	The design and key features of the evaluation, (cross referencing to any appropriate methodology reporting standards) and any changes to study protocol, with reasons		
Context	**7**	Additional file 2	The context in which the intervention was implemented. (Consider social, economic, policy, healthcare, organisational barriers and facilitators that might influence implementation elsewhere).		
Targeted 'sites'	**8**	Additional file 2	The characteristics of the targeted 'site(s)' (e.g locations/personnel/resources etc.) for implementation and any eligibility criteria.	N/A	The population targeted by the intervention and any eligibility criteria.
Description	**9**	Pgs 11-12	A description of the implementation strategy	Pgs 29-33 + Additional file 6	A description of the intervention
Sub-groups	**10**	N/A	Any sub-groups recruited for additional research tasks, and/or nested studies are described		
**Methods: evaluation**					
Outcomes	**11**	Pgs 11-12	Defined pre-specified primary and other outcome(s) of the implementation strategy, and how they were assessed. Document any pre-determined targets	N/A	Defined pre-specified primary and other outcome(s) of the intervention (if assessed), and how they were assessed. Document any pre-determined targets
Process evaluation	**12**	Additional file 6	Process evaluation objectives and outcomes related to the mechanism by which the strategy is expected to work		
Economic evaluation	**13**	N/A	Methods for resource use, costs, economic outcomes and analysis for the implementation strategy	N/A	Methods for resource use, costs, economic outcomes and analysis for the intervention
Sample size	**14**	Pgs 13-14 (Study A) + Pgs 15-16 (Study B) + Pgs.16-17 (Study C)	Rationale for sample sizes (including sample size calculations, budgetary constraints, practical considerations, data saturation, as appropriate)		
Analysis	**15**	Pgs 13-14 (Study A) + Pgs 15-16 (Study B) +	Methods of analysis (with reasons for that choice)		

		Pgs.16-17 (Study C)			
Sub-group analyses	**16**	N/A	Any a priori sub-group analyses (e.g. between different sites in a multicentre study, different clinical or demographic populations), and sub-groups recruited to specific nested research tasks		
**Results**					
Characteristics	**17**	Pgs 13-14 (Study A) + Pgs 15-16 (Study B) + Pgs.16-17 (Study C)	Proportion recruited and characteristics of the recipient population for the implementation strategy	N/A	Proportion recruited and characteristics (if appropriate) of the recipient population for the intervention
Outcomes	**18**	Pg 22-29 (behaviour diagnosis)	Primary and other outcome(s) of the implementation strategy	N/A	Primary and other outcome(s) of the Intervention (if assessed)
Process outcomes	**19**	Pg 29-33 (intervention mapping) + Additional file 6	Process data related to the implementation strategy mapped to the mechanism by which the strategy is expected to work		
Economic evaluation	**20**	N/A	Resource use, costs, economic outcomes and analysis for the implementation strategy	N/A	Resource use, costs, economic outcomes and analysis for the intervention
Sub-group analyses	**21**	N/A	Representativeness and outcomes of subgroups including those recruited to specific research tasks		
Fidelity/ adaptation	**22**	Pgs 34-35 (Strengths/ Limitations)	Fidelity to implementation strategy as planned and adaptation to suit context and preferences	N/A	Fidelity to delivering the core components of intervention (where measured)
Contextual changes	**23**	N/A	Contextual changes (if any) which may have affected outcomes		
Harms	**24**	N/A	All important harms or unintended effects in each group		
**Discussion**					

Structured discussion	**25**	Pgs 35-40 + Additional file 5	Summary of findings, strengths and limitations, comparisons with other studies, conclusions and implications		
Implications	**26**	Pgs 40-42	Discussion of policy, practice and/or research implications of the implementation strategy (specifically including scalability)	N/A	Discussion of policy, practice and/or research implications of the intervention (specifically including sustainability)
**General**					
Statements	**27**	Pg 18 + Pgs 43-45	Include statement(s) on regulatory approvals (including, as appropriate, ethical approval, confidential use of routine data, governance approval), trial/study registration (availability of protocol), funding and conflicts of interest		

Transparency criteria for replicability, as described by Aguinis and Solarino (2019)

Criterion	Study A: Document analysis and focus groups with in-training midwives	Study C: Participatory Learning Workshop with practicing midwives
1. Qualitative method	Action Research. Pragmatism	
	Document analysis of written response to open-ended question followed by focus group discussion for clarification of initial COM-B classification/confirmation of coding based on more detailed TDF	Participatory learning workshop with modified Nominal Group Technique to gather and classify suggestions under a broad COM-B classification
2. Research Setting	University setting: Department of Nursing.<br><br>Important background information about setting:<br><br>• One of only two academic training programmes in Midwifery (postgraduate qualification post-Nursing degree)<br>• Reduced intake every two years, despite shortage of trained midwives, due to difficulties in practice placements (non-midwifery model of care) and challenges in timely completion of formal professional registration requirements due to high rate of Caesarean sections and medicalization of birth	Registered Midwives, as part of Continuous Professional Development activity offered by University<br><br>Important background information about setting:<br><br>• Fragmented maternal-child health care services between private (staff shortages/ 70% of births) and public sector<br>• Substantial difference in autonomy of Midwives between public (more) and private sector (less)<br>• Ongoing General Health System historic reforms (since 2020), including the 'unification' of private-public sectors, through the establishment of the National Health Insurance fund<br>• Politically motivated delays in enforcement of current legislation on minimum staff requirements in private maternity clinics<br>• Limited direct access to midwives (currently only six, by referral from a Gynecologist-Obstetrician) and limited range of services provided by Midwives reimbursed by the newly established General Healthcare System (GeSY).

3. Position of researchers along insider-outsider continuum	Core research team include academic instructors in Midwifery programme in Cyprus and researchers in the field of maternal-child health and health care services. Extended research team in “Baby Buddy Forward” project: Midwifery and Public Health instructors and researchers from Cyprus, Greece, Germany and the UK. Both core as well as wider research team represent range of disciplines: Midwifery, Nursing, Psychology, Public Health, Health Services Research, Adult education, Digital Health.	
	More *Insider*, in terms of the relationship and experience of the informants, due to the teacher-student relationship, as well as overall understanding of current issues of Midwifery in Cyprus. But, limited control over students’ clinical learning experiences due to centralized clinical placement mentoring system by the Ministry of Health/ Midwifery Council	More *Outsider*, to informants and work-place experiences, as well as the range of organizations they represent. Relationship with organizations is restricted to clinical placements of students in maternity clinics/ hospitals, with no control or direct influence over Maternal-Child Health Care services
4. Sampling procedures	Purposive sampling. Cohort of in-training midwives at the Cyprus University of Technology - one of only two such programmes nationally, each with a cohort of 10-12 students	Convenience sampling. Voluntary participation of sample of delegates to the Baby Buddy Cyprus webapp launch event. Open event, which attracted over 120 delegates (about one in three of midwifery workforce in Cyprus).
5. Relative importance of the participants	All newly-qualified Registered Nurses, one semester prior to graduation and registration by the Cyprus Nurses and Midwives Council. During their academic training, they are exposed to a variety of settings (both in the public as well as the private sector) as part of their clinical placements	All Registered Midwives, from all five state hospitals and several private clinics across the island, with variable work experience. Even though accreditation with Continuous Education Credits, which is a necessary requirement for renewal of re-registration, may have functioned as an incentive for large participation in the event, workshop participants may represent a particularly motivated group.
6. Documenting interactions with participants	Written response to an open-ended question, followed by recorded and transcribed focus group discussion based on a semi-structured topic guide of three core questions (along the COM-B domains) and prompt questions tapping on related TDF concepts	Modified Nominal Group Technique with 4 stages: (1) Silent generation of ideas in small-groups, (2) documenting as a group views and opinions on worksheet, classified along COM-B domains, (3) sharing and elaborating on suggestions with the rest of the group in round-robin session, (4) gathering suggestions on whiteboard and debating, where necessary, COM-B classification. No voting or ranking of

		factors by importance was incorporated in the process as the purpose was to collect as many suggestions as possible.
7. Saturation point	Data saturation was achieved since all transcribed material was coded, indexed and charted along COM-B/ TDF, with no left-over text. Furthermore, the focus groups discussion presented the opportunity to both enrich and verify findings. However, the data were only content analyzed deductively, as per the purpose of the study, whereas, an inductive or abductive content analysis approach may have produced different interpretations and new insights.	Data saturation was reached when there were no additional suggestions in the round-robin session which gave the opportunity for all participants/groups to contribute to the discussion in a structured manner. Nevertheless, theoretical saturation may have not been achieved due to the voluntary participation in the workshop (Vs a more heterogeneous sample) and the data collection method (Vs in-depth interviews).
8. Unexpected opportunities, challenges and other events	On-going historic reforms of the National Health Care System, since 2020, which brought to the forefront of the political discussion several long-standing issues regarding the status or midwifery in Cyprus, including maternity clinic staffing regulations, direct access to midwives without referral, autonomous practice and so on	
	N/A	The NGT classification was presented at a final plenary session to all delegates of the launch event, providing an opportunity to verify the resonance of the representation of the phenomenon (challenges to professional role and behaviour) with delegates who did not attend the workshop, thus cultivating group reflection.
9. Management of power imbalance	Even though participants were midwifery students in-training, the data collection process (i.e. written response followed by focus group) did not emphasize the student-teacher relationship. In fact, the discussion was facilitated by a Public Health/ Health Services Researcher (not a midwife/ clinician), and hence, a novice in terms of the actual experience of the midwifery clinical practice. None of the questions were sensitive, and in fact did not related to 'self' (which may have exacerbated feelings of blame) but to 'midwives'	The participatory nature of the process passed the power to the participants. The researchers were only facilitating and structuring the process. The classification of factors into COM-B domains was self-directed and facilitators would provide guidance (by asking prompt questions) for the group to discuss debate and assign a suggestion to one of the three columns based on group consensus (even if researchers felt that the description may tap on more that one domain)

	in general. It is indicative that most quotes refer to what 'others' and 'some midwives' do, representing a reflection of practice through the eyes of midwifery students.	
10. Data coding and first-order codes	Deductive (directed) content analysis as more fitting both to the study purpose of the study and data collection method. The framework included a priori categories based on the COM-B and TDF. A set of codes was prepared based on the operational definition of each domain and developed iteratively along related concepts. The material was coded, indexed and charted accordingly along the COM-B and TDF model, with no left-over meaningful text. The process was led by two researchers independently from each other, who discussed and debated frequently. Any disagreement was settle by consensus, involving where necessary, a third researcher. The findings were presented to the wider team in the context of designing the intervention, who identified the readability of the narrative, the reasonableness of the interpretation as well as the relevance of the representation which informed the selection of relevant intervention components	The classification along the dimensions of Capacity, Opportunity and Motivation was proposed during the self-directed process led by the participants themselves during the workshop. When participants found it difficult to classify their suggestions according to COM, the facilitators would assist the group to discuss, debate and decide which domain to best classify it under, with the understanding that some factors represent horizontal themes.
11. Data analysis and second- and higher-order codes	The analysis focused on manifest rather than latent structure, opting for low abstraction level and low interpretation degree. This was more appropriate given the data collection approach (short written responses and focus group discussions Vs richer descriptions from personal interviews) as well as the purpose of the study (formative research for proposing an intervention Vs in-depth understanding of the experiences of midwives in their role as antenatal educator). An inductive or	Manifest analysis, with no abstraction level and no interpretation. The analytical process did not entail any re-classification or further analysis of concepts. The findings from the workshops offered insights into the nature and sources of the challenges, including more tangible suggestions for improvement. These provided a more nuanced understanding of the issues, contributed to the interpretation of the overall findings and were integrated into the narrative. They also informed the selection of relevant intervention functions.

	abductive content analysis approach may have produced different interpretations and new insights.	
12. Data disclosure	Transcripts and video recording	Unprocessed worksheets from the workshops. Documents of suggestions list (by group and integrated across all groups) of classification matrix
