# Supplementary material for: Identifying barriers to the educational role of midwives in Cyprus and defining determinants in behaviour terms using the Behaviour Change Wheel: a mixed-method formative study
Source: BMC Health Serv Res. 2022 Oct 5;22:1233. doi: 10.1186/s12913-022-08599-7 (PMC9534462; doi:10.1186/s12913-022-08599-7)
Supplement: Supplementary file 3 — Additional file 3. Open-ended question and semi-structured interview guide for the focus group session. [file 12913_2022_8599_MOESM3_ESM.pdf]

Additional File 3

Open-ended question and semi-structured interview guide for the focus group session.

Open-ended question

<u>Introduction:</u> The aim of prenatal education is not only to prepare for childbirth, but to help future parents stay healthy by enhancing knowledge, shaping attitudes and developing skills for their new role. It has been described as a "window of opportunity" for the promotion of health of mother-child-family. Antenatal education includes:

• Structured programmes, which commonly include a series of lectures, seminars and / or workshops most commonly in group sessions of various duration and content

• Information leaflets and other printed or digital material, commonly limited to providing information rather than developing practical skills, and

• Through personal contact with healthcare providers (gynaecologist-obstetrician, midwife) during routine appointments, which may nevertheless be characterized by limited time.

Question: As for the latter, which factors, in your opinion, facilitate (or would facilitate) and which factors make it difficult or present obstacles and barriers for a midwife to actively engage in this important role. You can express your thoughts on the matter and you may suggest as many factors as you like to discuss in our next session.

Semi-structured interview guide

- **Is antenatal education and counselling during routine appointments an activity that midwives commonly engage in? And if not, why?** You provided your thoughts on the matter in a written response. Can you please present your thoughts to the group and elaborate on your response?

- **Capability: Is it a matter of knowledge, skills and competences?**

Suggested prompt questions related to TDF, if not mentioned

- Do they know what they should do and how? Have they had **training**? (general, specific and procedural knowledge)
- Do they have the **skills** (physical, cognitive and/or interpersonal) to do it? (skills)
- Can they do it? What makes it easy and what hard? What **reminds** them to do it? (memory, attention and decision process)
- Is it easy to **plan** and set goals? Is it influenced by habit and normal routine? (memory, attention and decision process)
- How often does something else take higher **priority** or interfere with them engaging in this activity? (behavioural regulation)
- Are there procedures or ways of working that **encourages** them to engage in antenatal education? (behavioural regulation)

- **Opportunity: Would midwives by more/less likely to engage in antenatal education if certain aspects of the physical and/or social environment were different? So, is it a matter of the wider environment and context where this activity is taking place?**

Suggested prompt questions related to TDF, if not mentioned

- Which aspects or characteristics of the environment and work **context** influence whether or not they do it? (environmental context and resources)
- What material, equipment or other **resources** are needed to engage in this activity? Are they available? (environmental context and resources)
- What is the **norm**, the usual way this activity happens? Is the model way of engaging in this activity? (social influences)?
- How might views, opinions, perceptions or behaviours of **others** (colleagues, doctors, pregnant women, partners, professional associations, scientific bodies) influence their decision to do it? (social influences)
- What other **socio-cultural factors** are enabling or discouraging midwives to engage in this behaviour? (social influences)

- **<u>Motivation</u>: Is it a matter of beliefs and intentions? Would midwives be more likely to engage in antenatal education if they held more positive beliefs about the value of antenatal education or stronger intentions to engage in this role?**

Suggested prompt questions related to TDF, if not mentioned

- Who should be doing it? Is it part of their job and **professional role**? (social/ professional role & identity)
- Do they believe they can perform this activity? Are they **confident**? Any specific areas of difficulty? (beliefs about capabilities)
- Do they know why they need to do it? Do they believe it is **worthwhile** i.e. benefits for mother-baby-family or relationship with service users? Do they think there are **harms** in doing it/not doing it? (beliefs about consequences)
- Do they think it's something that **can be done**? (optimism)
- How do they feel about doing it or not doing it? Does engaging/or not engaging in this activity invoke an **emotional** response? (emotion)
- How strong are their **intentions**? How determined are they to claim this role? (intention)?
- Can they plan actions and set **goals**? Does something stand in the way? (goals)
- Are there **incentives**? Is there anything that might be **reinforcing** it? What is triggering the behaviour i.e. to remind them? (reinforcement)
