# Supplementary material for: Identifying barriers to the educational role of midwives in Cyprus and defining determinants in behaviour terms using the Behaviour Change Wheel: a mixed-method formative study
Source: BMC Health Serv Res. 2022 Oct 5;22:1233. doi: 10.1186/s12913-022-08599-7 (PMC9534462; doi:10.1186/s12913-022-08599-7)
Supplement: Supplementary file 4 — Additional file 4. Survey questionnaire referring to A=Action, C=context, T=time, and Ta=target with equivalent Greek translation of items referring to antenatal education and counseling [A] during routine antenatal appointments [C, T] with pregnant women and partners [Ta]. [file 12913_2022_8599_MOESM4_ESM.pdf]

Additional File 4:

Survey questionnaire referring to A=Action, C=context, T=time, and Ta=target with equivalent Greek translation of items referring to antenatal education and counseling [A] during routine antenatal appointments [C, T] with pregnant women and partners [Ta].

Domains (N=14))	Items	Source – see notes	Sub-domain (N=24)	Greek translation: main stem and items	
**Knowledge**	In my work with [intervention] I know exactly what is expected from me	(3)	**Knowledge, Procedural knowledge & Role clarity (4 items)**	Στο πλαίσιο της προγεννητικής εκπαίδευσης και συμβουλευτικής προς τις εγκύους, γνωρίζω ακριβώς τι αναμένεται από μένα	
	I am aware of how to [A] in [C, T] with [Ta]	(1)		Αναφορικά με την παροχή εκπαίδευσης και συμβουλευτικής προς τις εγκύους και τους συντρόφους τους στο πλαίσιο των προγραμματισμένων επισκέψεων (ραντεβού)...	…έχω υπόψη μου τις βασικές αρχές και τη διαδικασία
	I know how to [A] in [C, T] with [Ta]	(2)			…γνωρίζω τις βασικές αρχές και τη διαδικασία
	I am familiar with how to [A] in [C, T] with [Ta]	(2)			…είμαι εξοικειωμένη/ος με τις βασικές αρχές & τη διαδικασία
**Skills**	I have been trained how to [A] in [C, T] with [Ta]	(1)	**Skills (4 items)**		…έχω λάβει σχετική εκπαίδευση ή κατάρτιση
	I have the skills to [A] in [C, T] with [Ta]	(1)			…έχω τις απαραίτητες δεξιότητες
	I have practiced [A] in [C, T] with [Ta]	(1)			…έχω κάνει πρακτική εξάσκηση
	I have the proficiency to [A] in [C, T] with [Ta]	(2)			…έχω επάρκεια
**Optimism**	With regard to [A] in [C, T] with [Ta] in uncertain times, I usually expect the best	(1)	**Optimism (3 items)**		…συνήθως αναμένω το καλύτερο, ακόμα και σε καταστάσεις αβεβαιότητας
	With regard to [A] in [C, time] with [Ta] I'm always optimistic about the future	(1)			…είμαι πάντα αισιόδοξη/ος για το μέλλον
	With regard to [A] in [C, T] with [Ta] overall, I expect more good things to happen than bad	(2)			…αναμένω να υπάρξουν περισσότερες θετικές παρά αρνητικές εξελίξεις
**Social/professional role and identity**	[A] in [C, T] with [Ta] is part of my work as a [profession]	(1)	**Professional role (4 items)**	Η παροχή εκπαίδευσης και συμβουλευτικής στο πλαίσιο των προγραμματισμένων επισκέψεων (ραντεβού)...	…είναι μέρος της δουλείας μου ως μαία
	As a [profession], it is my job to [A] in [C, T] with [Ta]	(1)			…είναι επαγγελματική μου αρμοδιότητα (ως μαία)
	It is my responsibility as a [profession] to [A] in [C, T] with [Ta]	(1)			…είναι υποχρέωση μου ως μαία
	Doing [A] in [C, T] with [Ta] is consistent with my [profession]	(1)			…είναι σε συνάφεια με το επάγγελμα της μαίας
**Beliefs about capabilities**	I am confident that I can [A] in [C, T] with [Ta] even when [Ta] is not motivated	(1)	**Self-efficacy (3 items)**	Έχω αυτοπεποίθηση να παρέχω εκπαίδευση και συμβουλευτική στο πλαίσιο των ραντεβού…	…ακόμα και όταν οι ίδιες οι έγκυες δεν δείχνουν ενδιαφέρον
	I am confident that I can [A] in [C, T] with [Ta] even when there is little time	(1)			…ακόμα και όταν δεν υπάρχει πολύς χρόνος
	I am confident that if I wanted I could [A] in [C, T] with [Ta]	(1)			…αφού εάν θέλω, μπορώ να το κάνω

	How much control do you have over [A] in [C, T] with [Ta]? (No control at all – a lot of control)		(2)	**Perceived behavioural control (3 items)**	Πόσο έλεγχο έχεις της διαδικασίας παροχής εκπαίδευσης και συμβουλευτικής στο πλαίσιο των προγραμματισμένων επισκέψεων; (Καθόλου έλεγχο – Μεγάλο έλεγχο)	
	For me, [A] in [C, T] with [Ta] is… (Very difficult – very easy)		(2)		Για μένα προσωπικά, η παροχή προγεννητικής εκπαίδευσης και συμβουλευτικής στο πλαίσιο των προγραμματισμένων επισκέψεων είναι....	…πολύ δύσκολη υπόθεση– πολύ εύκολη υπόθεση
	For me, [A] in [C, T] with [Ta] is… (Impossible – possible)		(2)			…ανέφικτη – εφικτή
**Beliefs about consequences**	For me, [A] in [C, T] with [Ta] is… (Useless – useful)		(2)	**Attitudes (3 items)**		..είναι αχρείαστη – είναι χρήσιμη
	For me, [A] in [C, T] with [Ta] is… (Bad – good)		(2)			…είναι ανωφελής –είναι επωφελής
	For me, [A] in [C, T] with [Ta] is… (not worthwhile at all – very worthwhile)		(3)			… δεν αξίζει καθόλου τον κόπο – αξίζει τον κόπο πάρα πολύ
	If I [A] in [C, T] with [Ta] it will benefit	[Ta] ‘s health	(1)	**Outcome expectancies (5 items)**	Όταν παρέχω προγεννητική εκπαίδευση και συμβουλευτική προς τις εγκύους και τους συντρόφους τους...	…υπάρχουν οφέλη για την υγεία μητέρας-παιδιού
	If I [A] in [C, T] with [Ta] it will benefit	public health	(1)			…υπάρχουν οφέλη για τη δημόσια υγεία
	If I [A] in [C, T] with [Ta] it will have disadvantages for my relationship with [Ta]		(1)			…υπάρχουν κάποια μειονεκτήματα ως προς τη σχέση μου με τις εγκύους
	If I deliver [intervention] following guidelines, I feel satisfied		(3)			…αισθάνομαι ικανοποίηση
	If I deliver [intervention] following guidelines, this will strengthen the collaboration with professionals with who I deliver [intervention]		(3)			…ενδυναμώνει τη συνεργασία με άλλους επαγγελματίες υγείας
**Reinforcement***	Whenever I [A] in [C, T] with [Ta], I get financial reimbursement		(1)	**Reinforcement (4 items)**		…λαμβάνω οικονομική αποζημίωση
	Whenever I [A] in [C, T] with [Ta], I get recognition from professionals who are important to me		(1)			…λαμβάνω αναγνώριση από άλλους επαγγελματίες υγείας που εκτιμώ
	If I [A] in [C, T] with [Ta], I feel like I am making a difference (Never – always)		(2)			…αισθάνομαι ότι έχει νόημα και ότι κάνω τη διαφορά
	When I deliver [intervention] following guidelines, I get recognition from the participants		(3)			…είναι κάτι που αναγνωρίζεται θετικά από τις εγκύους και τους συντρόφους τους
**Intentions**	I intend to [A] in [C] with [Ta] in the next [T]		(1)	**Intentions (4 items)**	Στο επόμενο ραντεβού…	…θα προσπαθήσω να παρέχω εκπαίδευση και συμβουλευτική προς την έγκυο
	I will definitely [A] in [C] with [Ta] in the next [T]		(1)			…σίγουρα θα παρέχω εκπαίδευση και συμβουλευτική προς την έγκυο

	For how many of the next 10 [Ta] do you intend to [A] in [C]?	(1)		Σε πόσες από τις επόμενες 10 προγραμματισμένες επισκέψεις (ραντεβού), σκοπεύεις να παρέχεις προγεννητική εκπαίδευση και συμβουλευτική; (1-10)	
	How strong is your intention to [A] with [Ta] in [C] in the next [T]? (Not strong at all – Very strong)	(1)		Πόσο ισχυρή είναι η πρόθεση σου να παρέχεις προγεννητική εκπαίδευση και συμβουλευτική στο επόμενο προγεννητικό ραντεβού (Καθόλου ισχυρή – Πάρα πολύ Ισχυρή)	
**Goals**	I have a clear plan how I will [A] in [C, T] with [Ta]	(2)	**Priority (4 items)**	Αναφορικά με παροχή προγεννητικής εκπαίδευσης προς τις εγκύους στο πλαίσιο των προγραμματισμένων ραντεβού, έχω ξεκάθαρο πλάνο και προγραμματισμό ...	…για τον τρόπο με τον οποίο θα το κάνω
	I have a clear plan how often I will [A] in [C, T] with [Ta]	(1)			…πόσο συχνά θα το κάνω
	Generally, in [C, T] with [Ta], how often is covering something else on your agenda a higher priority than [A] (Never – Always)	(1)		Γενικά, πόσο συχνά κάτι άλλο κατά τη διάρκεια των προγραμματισμένων ραντεβού…	…καταλαμβάνει υψηλότερη προτεραιότητα από την παροχή προγεννητική εκπαίδευση και συμβουλευτική (Ποτέ – Πάντα)
	Generally, in [C, T] with [Ta], how often is covering something else on your agenda more urgent than [A]	(2)			…είναι πιο επείγον από την παροχή προγεννητική εκπαίδευση και συμβουλευτική (Ποτέ – Πάντα)
**Memory, attention and decision processes**	[A] in [C, T] with [Ta] is easy to remember	(2)	**Memory and Attention (5 items)**	Για μένα προσωπικά, είναι εύκολο να θυμάμαι να αναλαμβάνω εκπαιδευτικό ρόλο στο πλαίσιο των προγραμματισμένων ραντεβού με τις εγκύους;	
	How often do you forget [A] in [C, T] with [Ta]? (Never – Almost always)	(1)		Πόσο συχνά ξεχνάς να αναλαμβάνεις εκπαιδευτικό ρόλο στο πλαίσιο των προγραμματισμένων ραντεβού; (Πάντα- Σχεδόν ποτέ)	
	When I need to concentrate to [A] in [C, T] with [Ta], I have no trouble focusing my attention	(1)		Όταν πρέπει να επικεντρωθώ στην παροχή προγεννητικής εκπαίδευσης και συμβουλευτικής προς τις εγκύους...	…δεν έχω πρόβλημα να συγκεντρώσω την προσοχή μου σε αυτή τη δραστηριότητα
	When trying to focus my attention on [A] in [C, T] with [Ta], I have difficulty blocking out distracting thoughts	(1)			…Έχω δυσκολία να αποτρέψω άλλες σκέψεις που αποσπούν την προσοχή μου
	When concentrating on [A] in [C, T] with [Ta], I can focus my attention so that I become unaware of what's going on around me	(1)			…Μπορώ να εστιάσω σε αυτό ώστε να μην με ενοχλεί ο'τι συμβαίνει γύρω μου
**Environmental context and resources**	Within the socio-political context there is sufficient [financial] support (e.g., from local authorities, insurance companies, the government) for [innovation/guideline]	(1) /(3) for additional term	**Characteristic of the socio-political context (4 items)**	Αναφορικά με την παροχή εκπαίδευσης στο πλαίσιο των προγραμματισμένων	…υπάρχει επαρκής οικονομική υποστήριξη (π.χ. από τοπικούς φορείς, ασφαλιστικές εταιρείες, την κυβέρνηση)

		added in [ ]		ραντεβού και δεδομένου του υφιστάμενου κοινωνικοπολιτισμικού πλαισίου ή/και του οργανισμού που εργάζομαι...	
	Within the socio-political context there are good networks between parties involved in [innovation/guideline]	(1)			…υπάρχουν καλά δίκτυα και κανάλια επικοινωνίας μεταξύ των εμπλεκόμενων φορέων
	[Innovation/guideline] has a good fit with routine practice	(2)			…αυτή είναι συμβατή με την υφιστάμενη κλινική πρακτική
	In the organization I work [A] in [C, T] with [Ta] is routine	(2)			…αποτελεί μέρος της ρουτίνας
	In the organization I work there is enough time to [A] in [C, T] with [Ta]	(2)	**Organizational resources (6 items)**		…υπάρχει επαρκής χρόνος
	Prior to delivery of [innovation /guideline] professionals are provided with a training to [A] in [C, T] with [Ta]	(2)			…οι μαίες λαμβάνουν σχετική εκπαίδευση γι' αυτή τη δεξιότητα
	In the organization I work, all necessary resources are available to deliver [intervention]	(3)			…όλοι οι απαραίτητοι πόροι είναι διαθέσιμοι
	During the delivery of [innovation/guideline] professionals are provided with sufficient financial reimbursement to [A] in [C, T] with [Ta]	(2)			…υπάρχουν ικανοποιητικά οικονομικά ή άλλα κίνητρα
	[Implementing organization] provides sufficient [intervention] material	(3)			…είναι διαθέσιμο το απαραίτητο υλικό
	[Implementing organization] provides assistance to professionals with delivering [intervention]	(3)			…παρέχεται βοήθεια προς τις μαίες ώστε να αναπτύξουν αυτό το ρόλο
	Participants of [intervention] are motivated	(3)	**Characteristics of participants (2 items)**	Οι έγκυες γυναίκες και οι σύντροφοι τους ...	…δείχνουν ενδιαφέρον για την προγεννητική εκπαίδευση και συμβουλευτική
	Participants of [intervention] are positive about [intervention]	(3)			…είναι θετικοί ως προς την παροχή προγεννητικής εκπαίδευσης και συμβουλευτικής από τη μαία
**Social influences**	Most people who are important to me think that I should [A] in [C, T] with [Ta]	(1)	**Subjective norm (2 items)**	Οι περισσότεροι άνθρωποι που είναι σημαντικοί για μένα και των οποίων εκτιμώ τη γνώμη...	…πιστεύουν ότι πρέπει να παρέχω προγεννητική εκπαίδευση και συμβουλευτική στις εγκύους στο πλαίσιο των προγραμματισμένων επισκέψεων
	Most people whose opinion I value would approve me of [A] in [C, T] with [Ta]	(1)			…πιστεύουν ότι έχω επάρκεια να παρέχω προγεννητική εκπαίδευση και συμβουλευτική στο πλαίσιο των προγραμματισμένων επισκέψεων
	My colleagues are willing to listen to my problems related to [A] in [C, T] with [Ta]	(2)	**Social support (4 items)**	Οι συνάδελφοι μου...	…είναι πρόθυμοι να ακούσουν κάποιο πρόβλημα που είχα σχετικά με την παροχή προγεννητικής εκπαίδευσης και συμβουλευτικής προς εγκύους/συντρόφους

	I can rely on my colleagues when things get tough on [A] in [C, T] with [Ta]	(2)			…μπορώ να βασιστώ σε αυτούς όταν αντιμετωπίζω κάποια δυσκολία σε αυτό το ρόλο
	The team of professionals with whom I deliver [innovation] is helpful in getting [A] in [C, T] with [Ta] done	(2)		Η ευρύτερη διεπιστημονική ομάδα των επαγγελματιών υγείας με τους οποίους συνεργάζομαι ...	…είναι βοηθητικοί στο πλαίσιο της παροχής προγεννητικής εκπαίδευσης και συμβουλευτικής προς τις εγκύους και τους συντρόφους τους
	I can rely on the team of professionals with whom I deliver [innovation] when things get tough on [A] in [C, T] with [Ta]	(2)			…μπορώ να βασιστώ πάνω τους όταν αντιμετωπίζω κάποια δυσκολία που αφορά σε αυτό το ρόλο
	The team of professionals with whom I deliver [innovation/guideline] [A] in [C, T] with [Ta]	(2)	**Descriptive norm (2 items)**		…αναλαμβάνουν και αυτοί εκπαιδευτικό ρόλο κατά τα προγραμματισμένα ραντεβού
	Respected colleagues [A] in [C, T] with [Ta]	(2)		Οι συνάδελφοι μου...	…που πραγματικά εκτιμώ αναλαμβάνουν και αυτοί αυτόν τον εκπαιδευτικό ρόλο
	I can count on the support from the management of the organization I work in, when things get tough around delivering [intervention]	(3)	**Organizational support (3 items)**	Αναφορικά με τη διοίκηση του οργανισμού όπου εργάζομαι...	…μπορώ να βασιστώ πάνω τους όταν αντιμετωπίζω κάποια δυσκολία στο πλαίσιο της ανάληψης του εκπαιδευτικού ρόλου
	The management of the organization I work in is willing to listen to my problems with delivering [intervention]	(3)			…είναι πρόθυμοι να ακούσουν τα προβλήματα που υπάρχουν στο πλαίσιο της ανάληψης του εκπαιδευτικού ρόλου από τη μαία
	The management of the organization I work in is helpful with delivering [intervention]	(3)			…είναι υποστηρικτική ως προς την ανάληψης του εκπαιδευτικού ρόλου από τη μαία κατά τα προγραμματισμένα ραντεβού
**Emotion**	Have you recently, during the past two weeks been able to enjoy your normal day-to-day activities? (Never – Always)	(1)	**Stress (2 items)**	Κατά τις τελευταίες δύο εβδομάδες…	…ήσουν σε θέση να απολαύσεις τις συνήθεις καθημερινές σου δραστηριότητες; (Ποτέ – Πάντα)
	Have you recently, during the past two weeks been feeling unhappy and depressed? (Never – Always)	(1)			…αισθάνθηκες δυστυχισμένη και μελαγχολική; (Ποτέ - Πάντα)
	Thinking about yourself and how you normally feel as a professional that delivers [innovation/guideline], to what extent do you generally feel inspired with regard to [A] in [C, T] with [Ta] (Never- Always)	(2)	**Affect/ Positive & Negative emotions (2 items**	Σκεπτόμενη για το εαυτό σου, ως επαγγελματία υγείας που παρέχει προγεννητική εκπαίδευση, σε ποιο βαθμό αισθάνεσαι ότι αυτός ο ρόλος…	… είναι κάτι που γενικότερα σε εμπνέει (Ποτέ – Πάντα)
	Thinking about yourself and how you normally feel as a professional that delivers [innovation/guideline], to what extent do you generally feel nervous with regard to [A] in [C, T] with [Ta] (Never – Always)	(2)			…είναι κάτι που γενικότερα σου προκαλεί ανασφάλεια;
**Behavioral regulation**	[A] in [C, T] with [Ta] is something I do automatically	(2)	**Automaticity (4 items)**	Η παροχή εκπαίδευσης και	…Είναι κάτι που κάνω αυτόματα

	[A] in [C, T] with [Ta] is something I do without thinking	(2)		συμβουλευτικής στο πλαίσιο των προγραμματισμένων επισκέψεων (ραντεβού)...	…Είναι κάτι που κάνω χωρίς πολύ σκέψη
	Delivering [intervention] is something I do without having to consciously remember	(3)			…Είναι κάτι που κάνω χωρίς συνειδητή προσπάθεια να θυμηθώ να το κάνω
	Delivering [intervention] is something I start doing before I realize I am doing it	(3)			…Είναι κάτι που αρχίζω να κάνω χωρίς καν να αντιλαμβάνομαι ότι το κάνω
	I keep track of my overall progress towards [A] in [C, T] with [Ta]	(1)	**Self-monitoring (3 items)**	Αναφορικά με τις δικές σου συνήθεις και πρακτικές στο πλαίσιο παροχής προγεννητικής εκπαίδευσης….	… παρακολουθώ την πρόοδο μου
	I am aware of my day-to-day behavior as I work towards [A] in [C, T] with [Ta]	(1)			… έχω αντίληψη της καθημερινής μου πρακτικής
	I tend to notice my successes while working towards [A] in [C, T] with [Ta]	(2)			… έχω επίγνωση των επιτυχιών μου
	I have a clear plan with regards to delivering [intervention] when participants are not motivated	(3)	**Coping planning (3 items)**	Αναφορικά με παροχή προγεννητικής εκπαίδευσης προς τις εγκύους στο πλαίσιο των προγραμματισμένων ραντεβού, έχω ξεκάθαρο πλάνο και προγραμματισμό ...	…ακόμα και αν οι ίδιες οι γυναίκες δεν το αποζητούν
	I have a clear plan with regards to delivering [intervention] when there is little time	(3)			…ακόμα και αν δεν υπάρχει ικανοποιητικός χρόνος
	I have a clear plan with regards to delivering [intervention] when other professionals with whom I deliver [intervention] do not do this	(3)			…ακόμα κι αν οι άλλοι επαγγελματίες υγείας δεν το κάνουν

Notes: Adapted to include a total of 83 items across 14 domains and 24 sub-domains of the Theoretical Domains Framework, from three related sources (see below), numbered accordingly in the Table to reflect original source. The final Greek questionnaire includes all 36 items, suggested by Atkins et al (2017) based on discriminant validity of Huijg et al (2014b) as well as an additional 29 items and 18 items sourced from original 79-item by Huijg (2014b) and 93-item by Huijg (2014a) respectively in order to cover all 14 TDF domains with a substantial number of items.

Items appear in the order included in the questionnaire. For simplicity, items were organised in groups based on similarity and introduced in the questionnaire around a common stem. The response scale for all items is a 6-point Disagreement-Agreement Likert-scale, unless otherwise specified in the parenthesis.

Sources-References:

(1) Atkins L, Francis J, Islam R, O'Connor D, Patey A, Ivers N, Foy R, Duncan EM, Colquhoun H, Grimshaw JM, Lawton R. A guide to using the Theoretical Domains Framework of behaviour change to investigate implementation problems. Implementation Science 2017; 12(1): 77.

(2) Huijg JM, Gebhardt WA, Crone MR, Dusseldorp E, Presseau J. Discriminant content validity of a theoretical domains framework questionnaire for use in implementation research. Implementation Science 2014; 9(1): 11.

(3) Huijg JM, Gebhardt WA, Dusseldorp E, Verheijden MW, van der Zouwe N, Middelkoop BJ, Crone MR. Measuring determinants of implementation behavior: psychometric properties of a questionnaire based on the theoretical domains framework. Implementation Science 2014; 9(1): 33.
