# Supplementary material for: Identifying barriers to the educational role of midwives in Cyprus and defining determinants in behaviour terms using the Behaviour Change Wheel: a mixed-method formative study
Source: BMC Health Serv Res. 2022 Oct 5;22:1233. doi: 10.1186/s12913-022-08599-7 (PMC9534462; doi:10.1186/s12913-022-08599-7)
Supplement: Supplementary file 5 — Additional file 5. Rapid Literature Review: Barriers and enablers to midwives’ educational role as depicted in qualitative and/or quantitative studies that used the COM-B and/or the Theoretical Domains Framework. [file 12913_2022_8599_MOESM5_ESM.pdf]

Additional File 5

Rapid Literature Review: Barriers and enablers to midwives' educational role as depicted in qualitative and/or quantitative studies that used the COM-B and/or the Theoretical Domains Framework

Inclusion & Exclusion criteria:

- Investigated **barriers and enablers** to midwives' educational role irrespective of **focus** e.g. general health promotion or implementation of specific policy, programme, intervention and/or clinical guideline
- **Quantitative, qualitative and mixed-method** studies were considered
- Study sample either exclusively **midwives** or, if other health professionals included, midwives are also represented.
    - Studies focusing exclusively on other maternity care health professionals (GPs, health visitors etc) were not included.
    - Studies that explored barriers and facilitators as perceived by pregnant women were also not considered, unless separate reference provided to the views of midwives
- Either the **COM-B and/or the Theoretical Domains Framework** used to collect and/or analyse data as a structured means of behavior diagnosis
    - Studies included even if they did not proceed with selecting **intervention and/or policy functions and/or BCTs** (Behaviour Change Techniques) and possibly only discussed these in the form of implications in the Discussion section.

Search strategy:
Scopus, post 2000, articles in English

First search

midwife OR midwives OR midwifery
AND
antenatal OR prenatal OR perinatal OR pregnancy OR pregnant OR education OR counselling OR counseling OR "health promotion" OR implement OR implementation OR guidelines
AND
com-b OR "theoretical domain framework" OR tdf OR "Behavior Change Wheel" OR "Behaviour Change Wheel" OR bcw

Search hits: 19

Selected for further review from title/abstract: N=14

Excluded (N=5). List below with reason:

- Zinsser et al Midwifery (2020)
    - scoping review of components in behavior change interventions for pregnant women using the COM-B
- Flannery et al BMC Pregnancy and Childbirth (2018)
    - enablers and barriers to physical activity in overweight/obese pregnant women
- Rahimi et al Journal of Medical Internet Research (2018)
    - use of decision aid for down syndrome using Theory of Planned Behaviour (including some constructs of the Theoretical Domains Framework)
- Portocarrero et al BMC Pregnancy and Childbirth (2017)
    - factors influencing use of decision aid for prenatal screening for Down syndrome among pregnant women
- Olander et al Women and Birth (2016)
    - conceptual analysis of perinatal behavior change

Second search

midwife OR midwives OR midwifery OR antenatal OR prenatal OR perinatal
AND
com-b OR" theoretical domain framework" OR tdf OR" Behavior Change Wheel" OR "Behaviour Change Wheel" OR bcw

Search hits: 35

Selected for further review: N=18

Excluded (N=12), after removing duplicates excluded above (N=5). List below with reason:

- Esteves Mills et al BMC Public Health (2020)
    - systematic review of behaviour change interventions, behaviours or behavioural determinants during the perinatal period in LMIC
- Al Rawahi et al Oman Medical Journal (2020)
    - review of psychological models of health-related behavior in understanding sugars intake in adults
- Clarke et al Maternal & Child Nutrition (2020)
    - The ABA intervention for improving breastfeeding initiation and continuation. Sample: pregnant women (not midwives/health professionals)
- Reeks et al Australian Journal of Primary Health (2020)
    - Barriers and enablers to implementation of antenatal smoking cessation guidelines in general practice. Sample: GPs (not midwives)
- Walker et al BMC Family Practice (2019)
    - excess weight gain during pregnancy. Sample: GPs (not midwives)
- Holly et al Journal of Advanced Nursing (2019)
    - Midwives' own physical activity (not pregnant women)
- Grant et al BMC Pregnancy and Childbirth (2019)
    - Infant feeding intentions. Sample: pregnant women (not midwives/health professionals)
- Bar-Zeev et al BMC Pregnancy and Childbirth (2019)
    - Managing smoking in pregnancy. Sample GPs (not midwives)
- Muhwava et al Plos One (2019)
    - GDM. Sample: pregnant women (not midwives/health professionals)
- Ayoub et al BMC Medical Research Methodology (2018)
    - Pregnant women's participation in research
- Martis et al BMC Pregnancy and Childbirth (2018)
    - GDM. Sample: pregnant women (not midwives)
- McGoldrick et al BMC Pregnancy and Childbirth (2016)
    - Consumers attitudes and beliefs towards the receipt of antenatal corticosteroids and use of clinical practice guidelines

Table 1: Excluded after reading full text with reason (N=5)

(1) Haith-Cooper M, Stacey T, Bailey F, Broadhead-Croft S. The co-development and feasibility-testing of an innovative digital animation intervention (DAISI) to reduce the risk of maternal sepsis in the postnatal period. Maternal and Child Health Journal. 2020;24(7):837.
**Reason:** The study describes the process of user-testing a digital animation to reduce maternal sepsis. The COM-B model was used to analyse the findings in terms of user-testing the intervention among women from Black, Asian and Minority Ethnic groups. Even though midwives were included in the study, the findings are not presented in detail in the article.
(2) Bull ER, Hart JK, Swift J, Baxter K, McLauchlan N, Joseph S, Byrne-Davis LM. An organisational participatory research study of the feasibility of the behaviour change wheel to support clinical teams implementing new models of care. BMC health services research. 2019 Dec 1;19(1):97.
**Reason:** The study describes the experience of multidisciplinary clinical teams using the Behaviour Change Wheel to design, implement and evaluate a behaviour change intervention
(3) Agbadjé TT, Menear M, Dugas M, Gagnon MP, Rahimi SA, Robitaille H, Giguère AM, Rousseau F, Wilson BJ, Légaré F. Pregnant women's views on how to promote the use of a decision aid for Down syndrome prenatal screening: a theory-informed qualitative study. BMC health services research. 2018 Dec;18(1):1-5.
**Reason:** Study used the COM-B model to assess the relevance and acceptability among a sample of pregnant women of a series of suggested Behaviour Change Techniques to promote the use if a decision aid for Down syndrome screening, previously selected by the research team
(4) Heslehurst N, Newham J, Maniatopoulos G, Fleetwood C, Robalino S, Rankin J. Implementation of pregnancy weight management and obesity guidelines: a meta-synthesis of healthcare professionals' barriers and facilitators using the Theoretical Domains Framework. Obesity Reviews. 2014 Jun;15(6):462-86.
**Reason:** Study used the TDM in the context of secondary research/meta-synthesis of barriers and facilitators of implementation of pregnancy weight management and obesity guidelines
(5) Taylor NJ, Sahota P, Sargent J, Barber S, Loach J, Louch G, Wright J. Using intervention mapping to develop a culturally appropriate intervention to prevent childhood obesity: the HAPPY (Healthy and Active Parenting Programme for Early Years) study. International Journal of Behavioral Nutrition and Physical Activity. 2013 Dec;10(1):1-6.
**Reason:** Study used the TDF to classify determinants of childhood obesity based on needs assessment on the basis of literature.

Table 2: Included articles (N=15)

**Study number**	**Reference**
(1) Passey et al (2020)	Passey ME, Longman JM, Adams C, Johnston JJ, Simms J, Rolfe M. Factors associated with provision of smoking cessation support to pregnant women–a cross-sectional survey of midwives in New South Wales, Australia. BMC Pregnancy and Childbirth 2020;20: 1-0.
(2) Saronga et al (2020)	Saronga N, Burrows T, Collins CE, Mosha IH, Sunguya BF, Rollo ME. Nutrition services offered to pregnant women attending antenatal clinics in Dar es Salaam, Tanzania: A qualitative study. Midwifery 2020: 102783.
(3) Doherty et al (2020)	Doherty, E., Kingsland, M., Wiggers, J., Anderson, A.E., Elliott, E.J., Symonds, I., Tully, B., Dray, J. and Wolfenden, L.. Barriers to the implementation of clinical guidelines for maternal alcohol consumption in antenatal services: A survey using the theoretical domains framework. Health Promotion Journal of Australia: Official Journal of Australian Association of Health Promotion Professionals, 2020; 31(1): 133.
(4) Lucas et al (2019)	Lucas G, Olander EK, Salmon D. Healthcare professionals' views on supporting young mothers with eating and moving during and after pregnancy: An interview study using the COM-B framework. Health & Social Care in the Community 2020; 28(1): 69-80.
(5) McLellan et al (2019)	McLellan JM, O'Carroll RE, Cheyne H, Dombrowski SU. Investigating midwives' barriers and facilitators to multiple health promotion practice behaviours: a qualitative study using the theoretical domains framework. Implementation Science 2019; 14(1): 64.
(6) Wakida et al (2018)	Wakida EK, Obua C, Rukundo GZ, Maling S, Talib ZM, Okello ES. Barriers and facilitators to the integration of mental health services into primary healthcare: a qualitative study among Ugandan primary care providers using the COM-B framework. BMC Health Services Research 2018; 18(1): 890.
(7) Henshall et al (2018)	Henshall C, Taylor B, Goodwin L, Farre A, Jones ME, Kenyon S. Improving the quality and content of midwives' discussions with low-risk women about their options for place of birth: Co-production and evaluation of an intervention package. Midwifery 2018; 59: 118-26.
(8) Longman et al (2018)	Longman JM, Adams CM, Johnston JJ, Passey ME. Improving implementation of the smoking cessation

	guidelines with pregnant women: How to support clinicians?. Midwifery 2018; 58: 137-44.
(9) McParlin et al (2017)	McParlin C, Bell R, Robson SC, Muirhead CR, Araújo-Soares V. What helps or hinders midwives to implement physical activity guidelines for obese pregnant women? A questionnaire survey using the Theoretical Domains Framework. Midwifery 2017; 49: 110-6.
(10) Campbell et al (2017)	Campbell S, Roux N, Preece C, Rafter E, Davis B, Mein J, Boyle J, Fredericks B, Chamberlain C. Paths to improving care of Australian Aboriginal and Torres Strait Islander women following gestational diabetes. Primary Health Care Research & Development 2017; 18(6): 549-62.
(11) Nithianandan et al (2016)	Nithianandan N, Gibson-Helm M, McBride J, Binny A, Gray KM, East C, Boyle JA. Factors affecting implementation of perinatal mental health screening in women of refugee background. Implementation Science 2016; 11(1):150.
(12) Lepine et al (2016)	Lépine J, Portocarrero ME, Delanoë A, Robitaille H, Lévesque I, Rousseau F, Wilson BJ, Giguère AM, Légaré F. What factors influence health professionals to use decision aids for Down syndrome prenatal screening?. BMC Pregnancy and Childbirth 2016; 16(1): 262.
(13) Goldrick et al (2016)	Mc Goldrick EL, Crawford T, Brown JA, Groom KM, Crowther CA. Identifying the barriers and enablers in the implementation of the New Zealand and Australian Antenatal Corticosteroid Clinical Practice Guidelines. BMC Health Services Research 2016; 16(1): 617.
(14) Bain et al (2015)	Bain E, Bubner T, Ashwood P, Van Ryswyk E, Simmonds L, Reid S, Middleton P, Crowther CA. Barriers and enablers to implementing antenatal magnesium sulphate for fetal neuroprotection guidelines: a study using the theoretical domains framework. BMC Pregnancy and Childbirth 2015; 15(1): 176.
(15) Beenstock et al (2012)	Beenstock J, Sniehotta FF, White M, Bell R, Milne EM, Araujo-Soares V. What helps and hinders midwives in engaging with pregnant women about stopping smoking? A cross-sectional survey of perceived implementation difficulties among midwives in the North East of England. Implementation Science 2012; 7(1): 36.

Table 3: Study design characteristics and findings of identified studies

Study	Country/Setting	Focus	Study Design	Sample	Use of COM-B and/or TDF	Main findings	Intervention functions and/or BCTs
(1) Passey et al (2020)	New South Wales, Australia	Provision of smoking cessation support to pregnant women based on the 5As model (Ask, Advice, Assess, Assist, Arrange)	Online survey	150 midwives (20% response) from 14 LHDs invited to participate by Clinical Midwifery Consultants	40 item (final version) 5-point Likert scale questionnaire based on 12 domain TDF<br><br>Factor analysis to identify constructs	**Enablers**: Capability (knowledge, skills, confidence); Work environment (resources, capacity, champions and values); Personal priority (part of role and a priority)<br><br>**Barriers**: Intentions and Memory; Work environment; Negative perceptions	No explicit selection of intervention functions and/or BCTs<br><br>**Suggestions in discussion:**<br>• Skills training<br>• Local cessation champions<br>• Reframing perceptions of smoking from 'private issue' to consequences
(2) Saronga et al (2020)	Three unicipal hospitals, Dar es Salaam, Tanzania	Nutrition services to pregnant women in antenatal clinics	In-depth interviews	14 nurses (of whom 5 nurse midwives)* providing antenatal health services	Interview topic guide and content analysis based on 8 domains of the TDF	**Barriers and Enablers:** knowledge; skills; belief about capabilities; memory, attention and decision processes; environmental context and resources	No explicit selection of intervention functions and/or BCTs<br><br>**Suggestions in discussion:**<br>• In-service education and training

						**Enablers:** Social/ Professional role and Identity; Optimism; Beliefs about Consequences	• Resource material<br>• Staffing
(3) Doherty et al (2020)	Three sectors of one Local Health District, New South Wales, Australia	Implementation of clinical guidelines for maternal alcohol consumption in antenatal services	Online survey	33 antenatal clinicians (30% response) and 8 managers (50% response), of whom 23 and 5 midwives	56 item 5-point Likert scale questionnaire based on 11 domains of the TDF	**Barriers:** 7/6 domains identified as barriers by clinicians and managers respectively<br><br>**Lowest scores**: Environmental context and resources; Social influences; Beliefs about capabilities; Behavioural regulation (clinicians)	No explicit selection of intervention functions and/or BCTs<br><br>**Suggestions in discussion:**<br>• Enhancing service environment with prompts<br>• Localized procedures and pathways<br>• Educational meetings and materials<br>• Local opinion leaders and champions
(4) Lucas et al (2019)	UK	Supporting young mothers with eating and moving during and after pregnancy	Semi-structured interviews	17 midwives, family nurse practitioners and health visitors	Thematic analysis using the COM-B as a framework	**Capability:** specialist vs tacit knowledge<br><br>**Opportunity:** lack of time against	No explicit selection of intervention functions and/or BCTs

						difficult social context/ building trust<br><br>**Motivation:** part of role but difficult to prioritize. Lack of interest from women. Influenced by own body experiences and health behaviours	**Suggestions in discussion:**<br>• Communication skills training<br>• Enablement of time<br>• Reflection on own attitudes and beliefs
(5) McLellan et al (2019)	UK	Multiple health promotion practice behaviours (HePPBes)	Semi-structured interviews (study 1), followed by online questionnaire survey, with free text responses (study 2)	11 community midwives (study 1)<br><br>505 midwives (with complete responses) recruited via social media, forums and email lists (study 2)	Interview topic guide and direct content analysis based on 12 domain TDF<br><br>Online survey (no details or findings provided). Only free text responses	**Barriers**: clinical workload, cognitive resources, quality of relationship with pregnant women, lack of continuity of care, difficulty accessing appropriate training<br><br>Key **facilitator**: motivation to support pregnant women	No explicit selection of intervention functions and/or BCTs<br><br>No suggestions in discussion
(6) Wakida et al (2018)	Uganda	Integration of mental health services into primary healthcare	Semi-structured interviews	20 clinical officers, nurses and midwives from six	Interview topic guide and thematic analysis based on COM-B	**Capability:** inadequacy of knowledge; no current training;	No explicit selection of intervention functions and/or BCTs

				clinical centres		burdensome guidelines<br><br>**Opportunity:** limited resources; not many clients to require routine usage; no cues to use of guidelines<br><br>**Motivation:** not feeling self-reliant; lack of MH specialist; conflicting priorities; no regulatory measures	**Suggestions in discussion:**<br>• Belief in capabilities<br>• Guidelines in simplified format<br>• Prompts and cues at point-of-care<br>• Regulatory measures<br>• Local adaptation<br>• Specialized training in MH
(7) Henshall et al (2018)	UK	Quality and content of place of birth discussions	Focus groups followed by mixed-methods design and evaluation of intervention	6 focus groups with 38 midwives (first stage and 58/66 (second & third stage)	Framework analysis using the COM-B	**Capability:** lack of confidence in clinical skills regarding home birth and knowledge in providing information; uncertainty about "right language"; making assumptions about what women want; limited understanding of available services	Suggested a list of intervention functions and related BCTs which were evaluated in workshops with midwives.<br><br>A complete and detailed intervention package was co-produced and evaluated.

						**Opportunity:** inadequate resources; lack of exposure to homebirth; language and time barriers<br><br>**Motivation:** competing priorities; low value due to assumptions around women's interest of eligibility; model of care historical concerns/dedicated homebirth team; social and cultural norms impact on motivation	
(8) Longman et al (2018)	New South Wales, Australia	Provision of smoking cessation support to pregnant women based on the 5As model (Ask, Advice, Assess, Assist, Arrange)	Semi-structured interviews	27 maternity service managers, obstetricians and midwives from 14 LHD	Interview topic guide and thematic analysis based on 14 domain TDF	**Barriers:** no implementation or monitoring support systems; lack of knowledge, skills and training; perceived time restrictions; 'difficult conversations'; own perceptions around smoking.	No explicit selection of intervention functions and/or BCTs<br><br>**Suggestions in discussion:**<br>• Information and practice with evidence-based mechanisms for Assisting

						**Enablers**: beliefs about consequences; communication skills; professional role and identity; champions to influence practice; regulation of behaviour	• Understanding complexity of quitting process<br>• Beliefs about consequences and re-framing as 'addiction' rather than 'lifestyle choice'<br>• Training in motivational interviewing<br>• Leadership and policy to support consistency of messages<br>• Professional role and identify/ positive emotions<br>• Champions<br>• Behavior regulation through record keeping system
(9) McParlin et al (2017)	UK	Implementation of physical activity guidelines with obese pregnant women	Questionnaire survey	192 clinical midwives (53% response) from two hospital Trusts and	40 items with a 5-point Likert scale based on 11 domain TDF (nature of	**Highest scores:** Social professional role; knowledge<br><br>**Lowest scores:** skills; beliefs about capabilities;	No explicit selection of intervention functions and/or BCTs<br><br>**Suggestions in discussion:**

				community midwives from a third Trust	behaviour not included)<br><br>Also, free text comments analysed based on the TDF	behavior regulation. Also, resources and conflicting priorities.<br><br>**Skills and Memory, Attention & Decision making** strongest predictors of self-reported behaviour	• Training in communication skills and motivational interviewing<br>• Time, resources and referral powers or pathways<br>• Prompts, triggers and cues e.g. information leaflets<br>• Physical activity advice to all women to avoid potential stigma
(10) Campbell et al (2017)	Australia	Postpartum screening for type 2 diabetes following gestational diabetes in Indigenous women	Focus groups, including brainstorming, visualization, sorting and prioritizing activities.	7 Indigenous women with previous GDM and 20 Indigenous health workers to explore barriers and enablers (stage one). 24 health professionals, including	Thematic analysis using COM-B. Link to TDF no clear<br><br>Also, intervention/ policy mapping	Barriers identified by Indigenous women and health workers were classified under all 6 domains of the COM-B.<br><br>For example, inconvenience and dislike of test and health food expensive (environmental	28 interventions and policies to address barriers, 16 of which received a priority rating by at least 8 participants.<br><br>Highest intervention priority: environmental restructuring to holistic support and community-led process

				midwives (stage 2) involved in intervention mapping		context); culturally unfriendly service and lack of family support (social influence)	Highest policy priority: increase in Indigenous health staff and cultural training for non-Indigenous staff; Service provision
(11) Nithianandan et al (2016)	Australia	Implementation of perinatal mental health screening in women of refugee background	Semi-structured interviews	28 midwives, obstetricians, perinatal MH and refugee experts, interpreters<br><br>Also, 9 women with refugee background	Thematic analysis prior to identifying a framework, subsequently examined in relation to TDF (and Cultural Competence Conceptual Framework)	**Barriers:** knowledge (of screening tools); skills (cultural competence); professional roles (clarity around roles and continuity); beliefs about capabilities (lack of expertise); beliefs about consequences (stigma); environmental context (interpreters, good quality translated screening tools, private setting); social influences (continuity of care to build trust, 'go-to' person) and behavioural	Expert consensus to select BCTs for the 8 domains identified with suggested examples to support (a) healthcare providers and (b) women.<br><br>No clear mention of intervention functions.

						regulation (clear referral guidelines, feedback mechanisms)	
(12) Lepine et al (2016)	Quebec, Canada	Use of decision aid by HP to support shared decision making for Down syndrome prenatal screening	Face-to-face or phone semi-structured interviews after watching a 10-min video with two simulated prenatal follow-up consultations	36 (86% response) obstetricians, family physicians and midwives	Interview topic guide and content analysis, subsequently mapped onto constructs and domains of 12 domain TDF	35 (of total of 64) distinct factors reported by 20% or more participants mapped onto 10 of the 12 TDF.<br><br>**Six most frequent**: positive appraisal (beliefs about consequences; availability of aid and time constraints (environmental context and resources); colleagues' approval (social influences); relevant source of information (motivation and goals; not knowing any PtDAs (knowledge)	No explicit selection of intervention functions and/or BCTs<br><br>**Suggestions in discussion:**<br>• Tailor intervention to health professionals as influential factors differ.<br>• Perceived time constraints Vs shift in shared decision-making approach.<br>• Publicize proven benefits and endorsement by opinion leaders
(13) Goldrick et al (2016)	Three district health boards, Auckland, New Zealand	Implementation of antenatal Corticosteroid Clinical	Semi-structured interviews or	35 and 38 health professionals across four	Interview topic guide and questionnaire	**Enablers:** 11 beliefs mapping to 7 domains: Beliefs about consequences;	No explicit selection of intervention functions and/or BCTs

		Practice Guidelines	online questionnaire	groups including midwives randomized to be interviewed or complete an online survey	based on 14 domain TDF.<br><br>Questionnaire: open-ended questions<br><br>Direct content analysis and frequency counts, mapped to 14 domain TDF and classified as barriers or enablers	knowledge; social influences; environmental context and resources; beliefs about capabilities; social professional role and identity; behavioural regulation.<br><br>**Barriers:** 11 beliefs mapping to 5 domains: Beliefs about consequences; knowledge; social influences; environmental context and resources; social professional role and identity	**Suggestions in discussion:**<br>• Persuasive communication<br>• Rehearsal of behaviour<br>• Demonstration by peer expert<br>• Multidisciplinary approach<br>• Clarity of professional roles & responsibilities<br>• Implementation tools for local performance such as audit and feedback<br>• Practical education
(14) Bain et al (2015)	Women's and Children's Hospital, South Australia	Implementing antenatal magnesium sulphate for fetal neuroprotection guidelines	Semi-structured interviews with close and open-ended questions	24 and 21 randomly selected obstetric and neonatal consultants and trainees and midwives,	Content analysis using the 14 domain TDF as framework	**Barriers:** lack of awareness of protocol; complex administration processes; time pressures; unpredictability of preterm birth; discrepancies between	No explicit selection of intervention functions and/or BCTs<br><br>No suggestions for intervention functions in discussion

				two different time periods		consultants' opinions<br><br>**Enablers:** continuous education for staff and women at risk for very preterm birth; reminders and prompts (check lists, visual signage, alerts), simplified processes; influential colleagues	
(15) Beenstock et al (2012)	North East England	Engaging with pregnant women about smoking cessation	Paper-based questionnaire survey	364 after excluding delivery-unit midwives (of 589, response 43%)	47 items with a 5-point Likert scale based on 11 domain TDF (nature of behaviour not included)<br><br>Content validity, internal consistency and principal component analysis	**Lowest scores:** Beliefs about consequences; Environmental Context and Resources<br><br>"Propensity to act" – single factor derived from all domains explained 66% of variance and predictive of self-reported behaviour	No explicit selection of intervention functions and/or BCTs<br><br>**Suggestions in discussion:**<br>• Training on effectiveness of brief smoking cessation interventions<br>• CO monitors and leaflets<br>• Prompts
