# Supplementary material for: Identifying barriers to the educational role of midwives in Cyprus and defining determinants in behaviour terms using the Behaviour Change Wheel: a mixed-method formative study
Source: BMC Health Serv Res. 2022 Oct 5;22:1233. doi: 10.1186/s12913-022-08599-7 (PMC9534462; doi:10.1186/s12913-022-08599-7)
Supplement: Supplementary file 6 — Additional file 6. Behavioural Change Techniques (BCTs) to support the identified intervention functions. [file 12913_2022_8599_MOESM6_ESM.pdf]

Additional File 6

Behavioural Change Techniques (BCTs) to support the identified intervention functions.

Intervention function	Individual BCTs
**Enablement**	**3. Support. 3.2. Social support (practical):**
Baby Buddy offers tangible practical help in the performance of the educational role by increasing the means (i.e. against current lack of educational material) and reducing barriers (e.g. physical and linguistic). It can increase the *Capability* of midwives to perform the behaviour (beyond any additional training that may be needed to enhance procedural knowledge and strengthen related skills and competences) as well as the *Opportunity* to perform the role in suboptimal physical space and context (beyond any additional environmental restructuring that may be necessary to provide prompts and cues to engage in the behaviour more frequently and more systematically).	
**Environmental restructuring**	**7.Associations. 7.1. Prompts and cues:**
Baby Buddy's rich resources (video and other material) can be used independently from the platform as prompts and cues e.g. Baby Buddy videos playing in maternity clinic waiting rooms' monitors and/or daily messages/ FAQs printed as informational leaflets and posters.	
	**12.Associations. 12.5. Adding objects to the environment:**
Maternity clinics can be enhanced with Baby Buddy avatars, posters and cards at the point-of-care/ service to offer prompts for parents-to-be (bottom-up) to initiate a conversation around the topic as well as cues for midwives to engage in antenatal education (top-down)	
**Training**	**4. Shaping knowledge. 4.1. Instructions on how to perform behaviour:**
In-service practice training in engaging with pregnant women using Baby Buddy to structure the conversation, including Skills Development Training in identified areas of need (e.g. interpersonal and intercultural communication skills training led by the University.	
	**6. Comparison of behaviour. 6.1. Demonstration of the behaviour:**
Provide an observable sample of the performance of the behaviour e.g. demonstrate to midwives how to structure a conversation around a topic of interest using Baby Buddy to engage with service-users. This can be originally led by the University and subsequently by the trained Baby Buddy Champions.	

	**8. Repetition and Substitution. 8.1. Behavioural practice/ rehearsal:**<br>Prompt student and junior practicing midwives to practice and rehearse the performance of the behaviour with supervision in order to increase skill and habit.
	**8. Repetition and Substitution. 8.3. Habit formation:**<br>Identify the best context, place and timing (which may vary across individual maternity clinics) to prompt rehearsal and repetition of the behaviour, so that the context elicits the behaviour. For example, between medical test and waiting for the appointment with the doctor.
	**15. Self-belief. 15.1. Verbal persuasion about capability:**<br>Even though beliefs about capabilities to perform the role were not directly questioned by the midwives, they did recognize specific skills and competences that need strengthening (Training). Furthermore, attentional control was influenced by heavy workload demands while perceived behaviour control was weak, especially as other activities perceived by the system as more urgent often take priority. Reinforcing the self-belief that the behaviour can be performed, arguing against self-doubts and asserting they can succeed may be a necessary component of training programmes as well as role modelling of the behaviour.
**Incentivisation**	**2. Feedback. 2.2. Feedback on behaviour:**<br>Through the “train the trainer” programme, experienced midwives (Baby Buddy Champions) will monitor the performance of involved service providers and provide informative feedback.
	**2. Feedback. 2.2. Feedback on outcomes of behaviour:**<br>Baby Buddy in-app user stats can provide feedback to maternity clinics in terms of their performance in promoting the use of Baby Buddy.
	**10. Reward and Threat. 10.1 Material Incentive:**<br>Continuous Education accreditation for all Baby Buddy related training activities. This is highly valued as it is a requirement for the renewal of the professional registration to the Midwives Registry. Accreditation can function as an incentive for more midwives to express interest in joining the training and a material reward upon completion.
	**10. Reward and Threat. 10.4: Social reward:**<br>Develop a “Baby Buddy Educator” certification process awarded to midwives who complete the training and a “Baby Buddy Champion” recognition award to acknowledge the performance of midwives who show excellence in the antenatal educator role.

Modelling — **13. Identity. 13.1. Identification of self as role model:**

Experienced and well-respected by peers' midwives can provide a "credible role model". They can also be a significant asset as the first Baby Budd Champions in the "Train the Trainer" programme. Thus, it is important to identify "key people" across maternity clinics and cultivate their sense that their own behaviour can positively influence others. Experienced midwives can act as mentors and provide observable examples of 'good practice' both in real time as well as on video uploaded onto the Baby Buddy webapp platform.

6. Comparison of behaviour. 6.2. Social comparison:

This can take two forms. Firstly, drawing attention of junior midwives to the performance of the antenatal education role by experienced and well-respected midwives (role models) to allow comparison between their own performance and the 'optimal' performance. Furthermore, using in-app user stats, drawing the attention of specific maternity clinics of their performance compared to other maternity clinics. While, in-app user stats do not currently identify the specific healthcare provider of the registered users, comparative statistics at the District level can still offer a valuable social comparison for performance. Furthermore, monitoring the increase in registrations following in-hospital training sessions can act as positive reinforcement.

6. Comparison of behaviour. 6.3. Information about others' approval:

Provide information about what people think about the behaviour. This should include both (a) statements by relevant professional associations (i.e. Midwives Committee of the Cyprus Nurses and Midwives Association) about the central role a midwife holds in antenatal education and (b) testimonials promoted on Baby Buddy webapp and partners' social media platforms about the positive experience of pregnant women and their satisfaction with their midwife.

Persuasion — **9. Comparison of outcomes. 9.1. Credible source:**

Present verbal and visual communication from a credible source in favour of the behaviour. This could be Professional Associations and Opinion leaders (among practitioners) as well as influencers (from the community) which promote the central role a midwife in antenatal education (midwife as the 'educator') and the necessity of antenatal education, not as preparation for childbirth, but through the concept of the transition to parenthood as a life-changing "window of opportunity" for promoting the health and well-being of the whole family.

13. Identity. 13.2. Framing/ Reframing:

Midwives' beliefs about the benefits of antenatal education are strong. In fact, it is viewed as a core function of their professional role. Nevertheless, it might be useful to suggest the deliberate adoption

of a new perspective, focusing on its vital purpose as key in showcasing the autonomous role of the midwife. Furthermore, cognitive restructuring of current perceptions and potential misconceptions through persuasive communication may be needed. For example, re-framing the concept of antenatal education from a highly structured formal activity in physical space (i.e. the "antenatal class") for the preparation for childbirth (currently the norm), to promoting the concept of "*making every contact count*" and the transition to parenthood as a life-changing "window of opportunity" for promoting the health and well-being of the whole family. Furthermore, framing Baby Buddy as a solution to several of the perceived barriers such as "*time constraints",* "*online misinformation*", "*linguistic barriers*" and anticipated regrets stemming *from unclear role boundaries and potential inter-professional group power conflicts*. The latter can be particularly highlighted since Baby Buddy material was co-created in consultation with all national professional / scientific bodies.

13. Identity. 13.3. Incompatible beliefs:

In addition to cognitive restructuring through reframing the role of the midwife and the purpose of antenatal education, draw attention to discrepancies between current behaviour and self-image in order to promote reflection and create discomfort through cognitive dissonance. For example, draw attention to the discordance between current practice (in a highly medicalized birth environment) in comparison to the self-identification of midwives as proponents of the midwifery-model of care and advocates of natural birth and women's' rights to Respectful Maternity Care. Several of the principles of Respectful Maternity Care, which are highly valued by midwives, such as providing personalised care, building trusting relationships and forging cooperative alliances can all be achieved through strengthening further the antenatal education role of the midwives.
